# Targeting cPLA_2_ derived lipid hydroperoxides as a potential intervention for sarcopenia

**DOI:** 10.1038/s41598-020-70792-7

**Published:** 2020-08-18

**Authors:** Gavin Pharaoh, Jacob L. Brown, Kavithalakshmi Sataranatarajan, Parker Kneis, Jan Bian, Rojina Ranjit, Niran Hadad, Constantin Georgescu, Peter Rabinovitch, Qitao Ran, Jonathan D. Wren, Willard Freeman, Michael Kinter, Arlan Richardson, Holly Van Remmen

**Affiliations:** 1 Physiology Department, University of Oklahoma Health Sciences Center, Oklahoma City, OK USA; 2 Aging and Metabolism Research Program, Oklahoma Medical Research Foundation, Oklahoma City, OK USA; 3 Reynolds Oklahoma Center on Aging, University of Oklahoma Health Sciences Center, Oklahoma City, OK USA; 4 Oklahoma Center for Neuroscience, University of Oklahoma Health Sciences Center, Oklahoma City, OK USA; 5 Genes and Human Disease Research Program, Oklahoma Medical Research Foundation, Oklahoma City, OK USA; 6 Department of Pathology, University of Washington, Seattle, WA USA; 7 Department of Cell Systems and Anatomy, UT Health San Antonio, San Antonio, TX USA; 8 South Texas Veterans Health Care System, San Antonio, TX USA; 9 Oklahoma City VA Medical Center, Oklahoma City, OK USA

**Keywords:** Lipid peroxides, Lipidomics, Fluorescent dyes, Ageing, Physiology, Mitochondria

## Abstract

Defects in neuromuscular innervation contribute significantly to the age-related decline in muscle mass and function (sarcopenia). Our previous studies demonstrated that denervation induces muscle mitochondrial hydroperoxide production (H_2_O_2_ and lipid hydroperoxides (LOOHs)). Here we define the relative contribution of mitochondrial electron transport chain (ETC) derived H_2_O_2_ versus cytosolic phospholipase A_2_ (cPLA_2_) derived LOOHs in neurogenic muscle atrophy. We show that denervation increases muscle cPLA_2_ protein content, activity, and metabolites downstream of cPLA_2_ including LOOHs. Increased scavenging of mitochondrial H_2_O_2_ does not protect against denervation atrophy, suggesting ETC generated H_2_O_2_ is not a critical player. In contrast, inhibition of cPLA_2_ in vivo mitigates LOOH production and muscle atrophy and maintains individual muscle fiber size while decreasing oxidative damage. Overall, we show that loss of innervation in several muscle atrophy models including aging induces generation of LOOHs produced by arachidonic acid metabolism in the cPLA_2_ pathway contributing to loss of muscle mass.

## Introduction

The pathological age-related loss of skeletal muscle mass and function (sarcopenia) contributes to decreased quality of life and independence and increases the risk of injury and chronic disease^1,2^. Preventing or reducing the effects of sarcopenia could increase quality of life, reduce risk of comorbidity development, and save billions of dollars in healthcare costs annually. Designing therapeutic interventions will remain difficult until the complex pathways and processes underlying sarcopenia are identified^3^.

Studies supporting a loss of innervation with age and a link between denervation and decreased muscle fiber size suggest that sarcopenia is a form of neurogenic atrophy^4–6^. In addition to the loss of muscle mass, contractile force also declines with age^7^. Loss of muscle strength occurs more rapidly than can be explained by the loss of muscle mass alone, and evidence suggests that a reduction in muscle quality and neuromuscular junction signaling also play a role in the functional decline of aging muscle^8^. Our previous studies using mice lacking the cytosolic superoxide (O_2_^−^) scavenger CuZn superoxide dismutase (CuZn SOD, Sod1^***−/−***^) demonstrate that elevated oxidative stress leads to an accelerated sarcopenia in the *Sod1*^***−/−***^ mice that is characterized by denervation, muscle hydroperoxide production, and muscle atrophy^9–11^. Returning expression of CuZnSOD specifically to motor neurons of the *Sod1*^***−/−***^ mice prevented denervation, muscle hydroperoxide production, and atrophy, supporting a link between loss of innervation, hydroperoxides, and muscle atrophy^12^. We have also shown that loss of innervation to skeletal muscle directly induces basal hydroperoxide production from isolated mitochondria including both hydrogen peroxide (H_2_O_2_) and lipid hydroperoxides (LOOHs)^11^. The magnitude of this hydroperoxide increase is correlated with the extent of muscle atrophy in several neurogenic atrophy conditions including aging^11^. We identified generation of arachidonic acid (AA) by cytosolic phospholipase A_2_ (cPLA_2_) as a major source of LOOHs in denervation; however, whether increased hydroperoxide production contributes to neurogenic atrophy has not been determined^13^.

The goal of the current study is to define the role of hydroperoxides in neurogenic atrophy by (1) measuring the identity and source of released hydroperoxides (H_2_O_2_ vs LOOHs) and (2) testing whether inhibiting specific hydroperoxide generation in vivo can modulate downstream atrophy. We hypothesize that the increase in muscle hydroperoxides released following denervation is a causal component of neurogenic muscle atrophy and sarcopenia. To test this, we investigated the identity and source of hydroperoxide production from muscle fibers in several neurogenic atrophy conditions including aging, a mouse model of oxidative stress-induced atrophy *(Sod1*^***−/−***^*),* surgical denervation (sciatic nerve transection), and a mouse model (SOD1^G93A^) of Amyotrophic Lateral Sclerosis (ALS) using a combination of scavengers and small-molecule inhibitors. We also tested whether H_2_O_2_ or LOOHs are causal to neurogenic atrophy using genetic approaches to increase H_2_O_2_ scavenging and pharmacological interventions to inhibit cPLA_2_ as a potential source of LOOHs in the sciatic nerve transection model.

We report that neurogenic atrophy primarily induces muscle LOOHs through cPLA_2_ metabolism of AA and not mitochondrial H_2_O_2_. In vivo cPLA_2_ inhibition mitigates denervation atrophy, while H_2_O_2_ scavenging does not. We identify the cPLA_2_ pathway as a negative regulator of muscle mass in neurogenic atrophy and a potential target for therapeutic intervention in sarcopenia and other diseases of muscle wasting.

## Results

### Neurogenic atrophy induces muscle hydroperoxide production and atrophy

**Figure 1:**
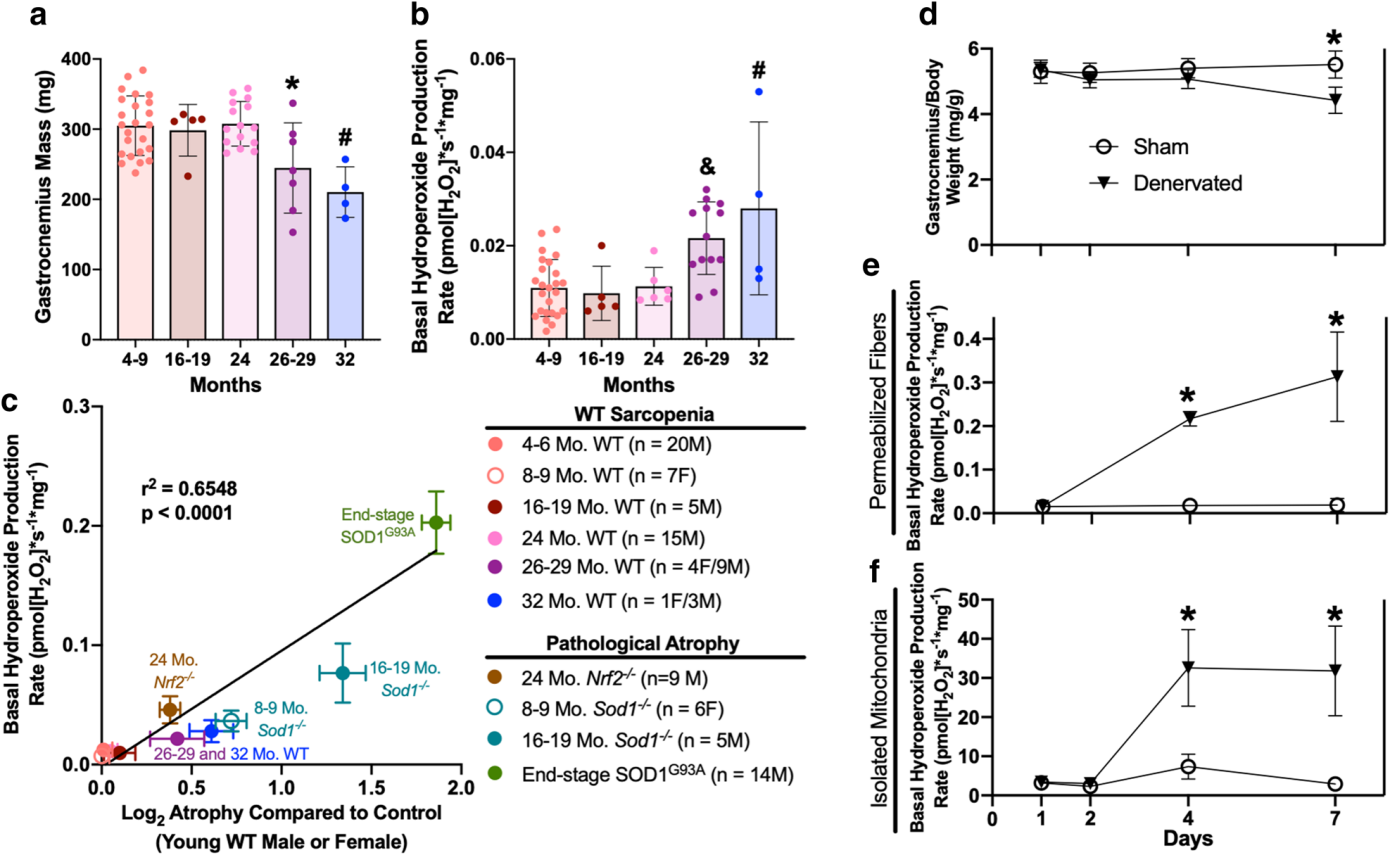
Neurogenic atrophy induces muscle hydroperoxide production and atrophy. (**a**) Combined gastrocnemius muscle mass (4–9 mo. WT n = 7F and 17 M; 16–19 mo. WT n = 5 M; 24 mo. WT n = 15 M, 26–29 mo. WT n = 4F and 3 M; 32 mo. WT n = 1F and 3 M) and (**b**) basal hydroperoxide production rate of permeabilized gastrocnemius fibers in both sexes of C57BL/6 J mice across a range of ages (4–9 mo. WT n = 7F and 20 M; 16–19 mo. WT n = 5 M; 24 mo. WT n = 7 M; 26–29 mo. WT n = 4F and 9 M; 32 mo. WT n = 1F and 3 M). Statistical significance determined by ordinary one-way ANOVA with tukey’s post hoc test (*p < 0.05 vs. 4–9 mo. WT and 24 mo. WT; &*p* < 0.05 vs. 4–9 mo. WT and 16–19 mo. WT; #*p* < 0.05 vs. 4–9 mo. WT, 16–19 mo. WT, and 24 mo. WT). (**c**) Basal hydroperoxide production rate of permeabilized muscle fibers versus the extent of atrophy relative to young female or male control. Statistical significance determined by linear regression. 4–6 month old male mice, 24 month WT, aged *Nrf2*^***−/−***^ and end-stage SOD1^G93A^ masses and hydroperoxide production rates were previously reported and included here for further comparison between multiple models^22,23^. Sham and denervated gastrocnemius (**d**) muscle mass (1 day n = 14; 2 days n = 4; 4 days n = 12; 7 days n = 35), (**e**) permeabilized fiber basal hydroperoxide production rate (1 day n = 6; 4 days n = 4; 7 days n = 18), and (**f**) isolated mitochondria basal hydroperoxide production rate (1 day n = 9; 2 days n = 4; 4 days n = 8; 7 days n = 6) in male mice. Statistical significance determined by ordinary two-way ANOVA with Sidak’s post hoc test (*p < 0.05 sham versus denervated at the same time point). Plots represent mean ± standard deviation, except plot C represents mean ± SEM.

Aging in mice and humans is associated with an age-related loss of motor neurons and sarcopenia^4,5^. Our previous studies show that loss of muscle mass in response to denervation is accompanied by increased mitochondrial generation of hydroperoxides^11,13^. To determine the effect of hydroperoxide production on the loss of muscle mass during aging, we compared gastrocnemius muscle mass and basal hydroperoxide production in permeabilized gastrocnemius muscle fibers harvested from young, middle aged, and old C57BL/6 J mice. Figure 1a shows a loss of gastrocnemius muscle mass evident first at 26 months of age that continues into advanced age (32 months), while Fig. 1b shows a clear association between basal hydroperoxide production rates and loss of gastrocnemius mass (Fig. 1a,b). The increase in basal hydroperoxide production rate correlates with the amount of muscle atrophy in aging mice and in several other advanced atrophy models, including mice lacking the Nrf2 antioxidant response transcription factor (*Nrf2*^***−/−***^), mice with accelerated neurogenic sarcopenia (*Sod1*^***−/−***^), and mice with motor neuron disease (SOD1^G93A^) (Fig. 1c). These data support a direct relationship between basal hydroperoxide production rate and neurogenic muscle atrophy.

To more directly interrogate the relationship between loss of innervation, muscle atrophy and hydroperoxide generation, we measured changes in hydroperoxide production rates and muscle mass in a model of sciatic nerve transection. This model is relevant to aging because the proportion of denervated fibers increases with age^6^. Denervation by surgical transection of the sciatic nerve was performed in one hindlimb (denervation) and compared to the effect of sham surgery in the contralateral hindlimb of adult, male C57BL/6 J mice. The sciatic nerve transection model allows us to measure time-dependent changes after loss of innervation in samples composed entirely of denervated fibers. We measured the muscle response to nerve transection at 1, 2, 4, and 7 days following surgery. Muscle mass declines significantly between 4 and 7 days after loss of innervation, while hydroperoxide production is increased between 2 and 4 days (Fig. 1d–f). By 7 days post denervation, muscle mass is decreased by 20% and hydroperoxide production rates are increased compared to sham in both permeabilized muscle fibers (1,468%) and isolated mitochondria (987%). These results are supported by a previous finding that basal hydroperoxide production is increased in denervated tibialis anterior (TA) muscle prior to atrophy^14^.

We also compared sex-specific effects of denervation on gastrocnemius muscle from female and male mice seven days after surgery. Gastrocnemius muscle from both sexes shows a significant decline in mass at 7 days post-denervation (Supplemental Fig. 1a). Male mice have a higher initial gastrocnemius mass and a greater loss of total muscle mass; however, the relative loss of mass is equivalent in both sexes (Supplemental Fig. 1b-c). The greater loss in muscle mass in male mice corresponds to higher hydroperoxide production rates in isolated mitochondria and permeabilized fibers from denervated muscles in male mice compared to female mice (Supplemental Fig. 1d-e).

### Loss of innervation induces transcriptional changes

**Figure 2:**
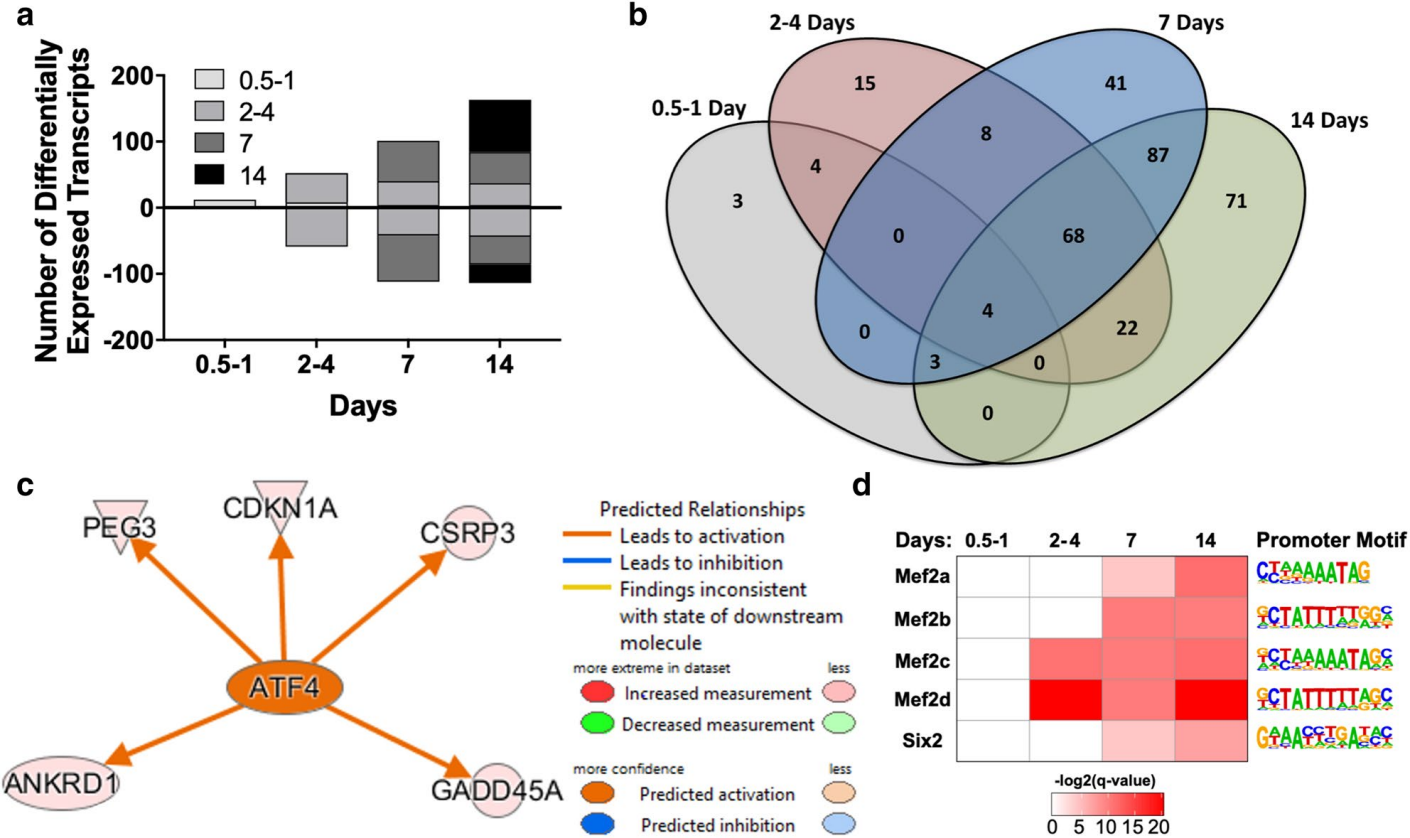
Loss of Innervation Induces Transcriptional Changes. (**a**) Differentially up- and down-regulated genes at each day relative to sham. Grey-scale colors represent which day the gene was initially significantly differentially regulated. (**b**) Venn diagram showing overlap of differentially regulated genes at each time point relative to sham. (**c**) Upstream regulator of gene expression changes determined using Ingenuity Pathway Analysis. (**d**) Significant enrichment of Mef2 isoform target genes beginning at 2–4 days relative to sham. All mice were male (n = 4 per time point). ANKRD1—Ankyrin Repeat Domain 1; ATF4—Activating Transcription Factor 4; CDKN1A—Cyclin Dependent Kinase Inhibitor 1A; CSRP3—Cysteine And Glycine Rich Protein 3; GADD45A—Growth Arrest and DNA Damage Inducible Alpha; Mef2—Myocyte Enhancer Factor 2; PEG3—Paternally Expressed 3; Six2—SIX Homeobox 2.

To understand the molecular events underlying the effects of denervation, we measured denervation induced gene expression changes in muscle using RNAseq analysis of denervated muscle samples across a time course following sciatic nerve transection. We found that an acute gene expression response to denervation begins within 12–24 h followed by a chronic response beginning between 2 and 4 days, which coincides with the observed increase in LOOH production by the cPLA_2_ pathway (Fig. 2a). Further changes in gene expression increase up to 14 days (Fig. 2a,b). A similar number of genes are upregulated compared to the number that are downregulated at each time point during the chronic response from days 2 through 14 (Fig. 2a). Four genes are significantly upregulated throughout the entire time course (*Arpp21*, *Gadd45a*, *Gdf5*, and *Myog*), and 68 of the genes that are differentially regulated beginning at 2–4 days persist throughout the measured chronic phase (Fig. 2b). By day 14, 35 biological processes (including processes related to muscle cell function, metabolism, intracellular signaling, and ion transport) are significantly affected through overrepresented differentially expressed genes (Supplemental Files). 88 canonical pathways show significant change in activity including activation of Production of Nitric Oxide and Reactive Oxygen Species in Macrophages and ERK/MAPK Signaling and significantly different expression of Calcium, AMPK, NRF2 Antioxidant Transcription Factor, p53, and GADD45A signaling pathways (Supplemental Files). Signaling from ATF4 as an upstream regulator of gene expression changes was elevated within 2–4 days (Fig. 2c). ATF4 was previously associated with age-related muscle atrophy but our findings now suggest that loss of innervation to muscle fibers plays a key role in inducing this pathway^15^. Of the differentially regulated genes, we observed a significant enrichment of genes containing transcription factor binding sites in their promoter regions for MEF2 beginning at 2–4 days and SIX2 at 7 days (Fig. 2d). In addition to upregulation of LOOH production, loss of innervation is associated with sweeping chronic changes in gene expression.

### Loss of innervation induces LOOH production through activation of the cytosolic phospholipase A_2_ (cPLA_2_) pathway

**Figure 3:**
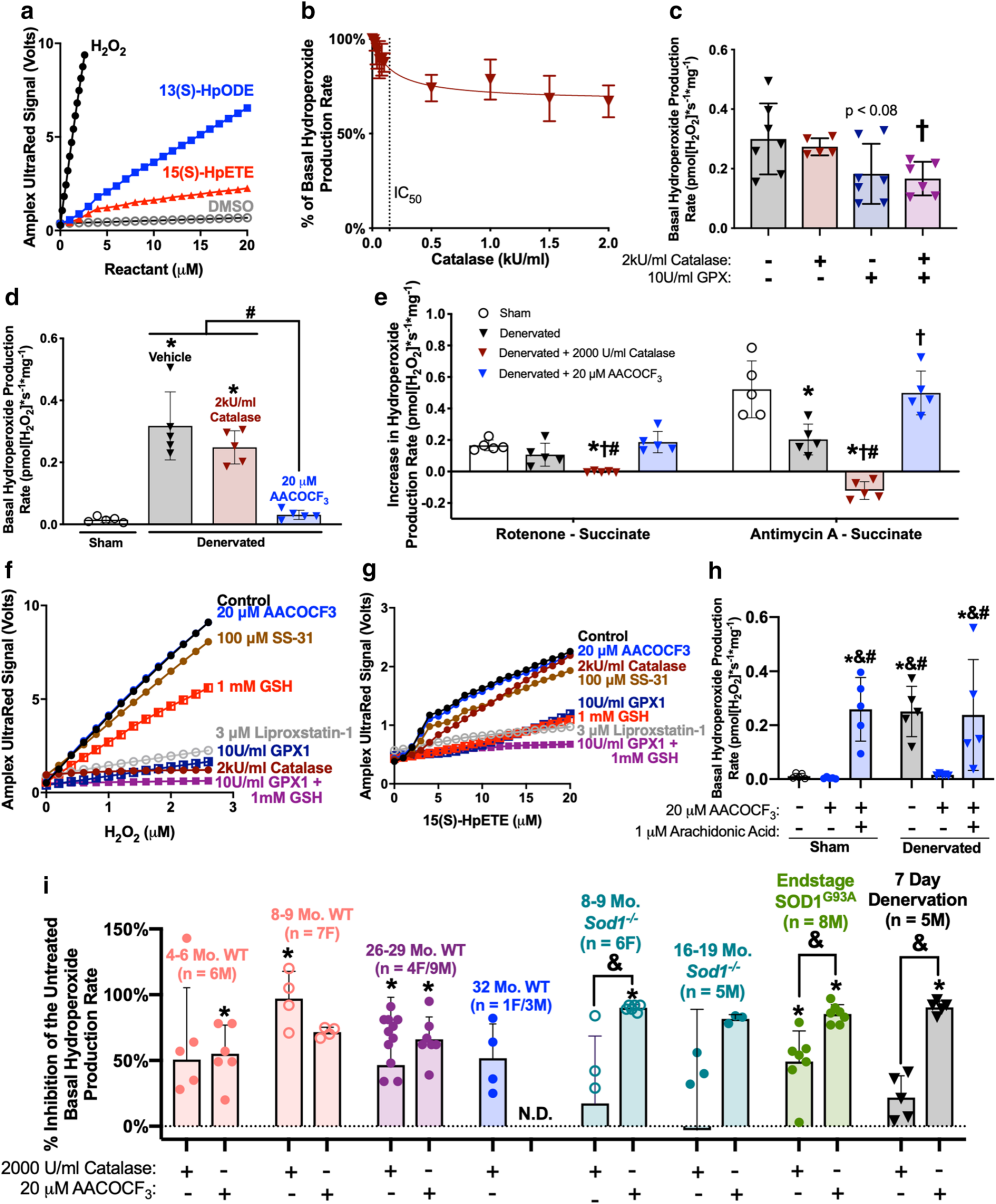
Loss of innervation primarily induces LOOH production through metabolism in the cytosolic phospholipase A_2_ (cPLA2) Pathway. (**a**) Dose–response of Amplex UltraRed fluorescence values in response to increasing concentrations of H_2_O_2_ or the LOOHs 15(S)-HpETE or 13(S)-HpODE. (**b**) Preliminary catalase inhibition dose–response curve in 7 day denervated muscle fibers (n = 2F). IC_50_ determined by variable slope (four parameters) test. (**c**) Basal hydroperoxide production rate in 7 day denervated fibers treated with catalase and/or GPX1 (n = 5–7 M). Statistical significance determined by ordinary one-way ANOVA with Tukey’s post hoc test. (**d**) Basal hydroperoxide production rate and (**e**) ETC inhibitor-induced hydroperoxide production rates in 7 day sham and denervated fibers treated with catalase or AACOCF_3_ (n = 5 M). Statistical significance determined by ordinary one-way ANOVA with Tukey’s post hoc test for each condition. Dose–response of Amplex UltraRed raw fluorescence values in response to increasing concentrations of (**f**) H_2_O_2_ or (**g**) 15(S)-HpETE in the presence of antioxidants and inhibitors at the specified concentrations. (**h**) Sham and denervated fibers treated with AACOCF_3_ and exogenous AA (n = 5 M). Statistical significance determined by ordinary two-way ANOVA with Tukey’s post hoc test. (**i**) Percent inhibition of basal hydroperoxide production rate in neurogenic atrophy models with catalase or AACOCF_3_ treatment relative to untreated controls for each model. Statistical significance determined by ordinary one-way ANOVA with Tukey’s post hoc test. For 16–19 Mo. *Sod1*^***−/−***^, n = 5 M total, except catalase inhibition was only performed on 4 samples and AACOCF_3_ inhibition was only performed on 3 samples due to limitations in the number of Oroboros O2k chambers available at the time of experimentation and thus was not powered to reach statistical significance. However, similar inhibition with AACOCF3 was observed compared to 8–9 month old Sod1^−/−^. End-stage SOD1G93A basal hydroperoxide production rate for vehicle and catalase or AACOCF3 inhibition was previously reported and included here for further comparison between multiple models23. *p < 0.05 versus vehicle sham or control; &*p *< 0.05 versus AACOCF3 sham or control; †*p *< 0.05 versus vehicle denervated; #*p *< 0.05 versus AACOCF3 denervated. All plots represent mean ± standard deviation. N.D.—No data.

We previously reported that in addition to detecting hydrogen peroxide (H_2_O_2_), the Amplex Red probe interacts with lipid hydroperoxides (LOOHs)^13^. Invitrogen has since released an updated probe (Amplex UltraRed, Invitrogen A36006) with improved sensitivity and stability. We report that H_2_O_2_ and the LOOHs 13(S)-HpODE and 15(S)-HpETE, two metabolites enzymatically produced in vivo by 12/15-lipoxygenase, react with Amplex UltraRed to produce a concentration-dependent linear increase in fluorescent resorufin^16^. Notably, H_2_O_2_ produces a stronger increase in fluorescence at the same concentrations (Fig. 3a).

Our previous study using isolated mitochondria revealed that inhibition of cytosolic phospholipase A_2_ (cPLA_2_) with arachidonyl trifluoromethyl ketone (AACOCF_3_), an inhibitor that binds to the cPLA_2_ active site to block its activity, prevented the increase in Amplex UltraRed signal in response to denervation^13,17^. However, the study by Bhattacharya et al. did not define the total hydroperoxide production in muscle fibers, whether LOOHs were being produced upstream or downstream of cPLA_2_, or whether cPLA_2_ produces LOOHs in other neurogenic atrophy models including aging. cPLA_2_ is a membrane associated enzyme that cleaves AA from membrane phospholipids, thereby increasing AA levels that are in turn converted by enzymatic pathways to eicosanoids, lipid mediators of cell signaling^18^. LOOHs can be released from membranes by cytosolic phospholipase A_2_ (**cPLA**_**2**_) or formed downstream of cPLA_2_ by metabolism of AA into LOOH intermediates^18–20^. To define the molecular identity of hydroperoxides produced by denervation using permeabilized muscle fibers, we used Amplex UltraRed and ex vivo treatment with a combination of antioxidants (catalase, glutathione peroxidase, and superoxide dismutase) and the cPLA_2_ inhibitor AACOCF_3_. All conditions contained 2.5 U/ml of exogenous CuZn superoxide dismutase (*Sod1*) to convert any superoxide (O_2_^−^) produced by the mitochondria to H_2_O_2_. First, to specifically scavenge H_2_O_2_ and not LOOHs produced downstream of cPLA_2_, we treated denervated muscle fibers with supraphysiological concentrations of exogenous catalase. Treatment of denervated fibers with catalase decreased the hydroperoxide production rate but only up to ~ 30%, even at supraphysiological concentrations of catalase (Fig. 3b). We next treated denervated fibers with exogenous glutathione peroxidase 1 (GPX1), which scavenges both H_2_O_2_ and LOOHs^21^. Combined treatment of denervated fibers with GPX1 and catalase significantly decreased the hydroperoxide production rate by approximately 50% (Fig. 3c). In contrast, the CPLA_2_ inhibitor AACOCF_3_ decreased the hydroperoxide signal to a much greater extent than catalase or GPX (approximately 90%) (Fig. 3d) suggesting the greater proportion of the Amplex UltraRed signal is generated by LOOH formed downstream of cPLA_2_ as opposed to H_2_O_2_.

To further define the source of the Amplex UltraRed signal in permeabilized fibers, we measured the effect of a series of titrations of mitochondrial electron transport chain (ETC) substrates and inhibitors on hydroperoxide production described in the methods. Adding the mitochondrial ETC inhibitors rotenone and antimycin A greatly increases hydroperoxide production (presumably as a result of O_2_^−^ and H_2_O_2_ from ETC complexes) in permeabilized muscle fibers^22,23^. We show here that both ETC inhibitors induced hydroperoxide production in both untreated sham and denervated fibers (Fig. 3e). Exogenous catalase and CuZnSOD completely scavenged the source of this signal while AACOCF_3_ had no effect (Fig. 3e). These results demonstrate that the CuZnSOD and catalase concentrations efficiently scavenge mitochondrial generation of O_2_^−^ and H_2_O_2_, and that AACOCF_3_ does not inhibit mitochondrial generation of O_2_^−^ and H_2_O_2_ or act as a direct antioxidant scavenger. Together, these data suggest that LOOHs are the primary component of the increased hydroperoxides observed after loss of innervation, their production or release requires cPLA_2_ activity, and they are not produced from electron transport chain reactive oxygen species (ROS) generation.

To determine the direct scavenging potential in vitro for a number of oxidant scavengers and AACOCF_3_, we generated a dose–response curve for H_2_O_2_ or the 12/15-lipoxygenase generated LOOH 15(S)-HpETE in the presence of several oxidative scavengers and AACOCF_3_ using the Amplex UltraRed signal as a readout^16^. Glutathione (GSH) is used to regenerate the antioxidant capacity of GPX1, although it can also serve as a direct antioxidant for free radicals, reactive oxygen species including H_2_O_2_, and LOOHs^24^. SS-31 is a mitochondrial targeted peptide that was originally designed as a mitochondrially-targeted antioxidant that directly scavenges via its tyrosine amino acid residue^25^. SS-31 was previously reported to completely inhibit H_2_O_2_ chemiluminescence at 100 µM and decrease lipid peroxidation by ~ 60% at 1 µM in vitro, although these experiments were carried out with isolated mitochondria or over an incubation period in cell-free conditions^25,26^. Liproxstatin-1 is a recently reported LOOH scavenger and functions as a positive control^27^. At the concentration we used in our permeabilized fiber assays, catalase and GPX1 with or without reduced glutathione (GSH) directly scavenge H_2_O_2_. In contrast, we observe no direct scavenging of LOOHs by AACOCF_3_ or SS-31 (Fig. 3f). We also report that Liproxstatin-1 functions as a scavenger of H_2_O_2_ in addition to LOOHs (Fig. 3f). For LOOHs, Liproxstatin-1, GPX1 with and without GSH, and GSH work as direct scavengers while SS-31 and AACOCF_3_ do not (Fig. 3g). AACOCF_3_ decreases LOOH production by inhibiting cPLA_2_ without direct scavenging of LOOHs.

The LOOHs produced in skeletal muscle during loss of innervation could be produced by two processes that are upstream or downstream of cPLA_2_: i.e., oxidative insult to membrane phospholipids creating LOOHs that are released by cPLA_2_, or metabolism of cleaved AA into eicosanoids that can produce LOOHs as a byproduct through the lipoxygenase and cyclooxygenase pathways^19,20^. We hypothesized that if denervated fibers exposed to cPLA_2_ inhibition by AACOCF_3_ treatment are able to produce hydroperoxides after addition of exogenous AA, this would be evidence that LOOH production is primarily occurring by metabolism of AA into eicosanoids. Exogenous addition of AA to sham and denervated muscle fibers treated with AACOCF_3_ increased hydroperoxide production rates to levels comparable to untreated denervation fibers (Fig. 3h). This suggests the cPLA_2_ pathway primarily produces LOOHs in denervated muscle downstream of cPLA_2_ release of AA and provides evidence that denervation-induced hydroperoxides are produced enzymatically during eicosanoid metabolism of AA and not by the canonical release of LOOHs from membranes formed by oxidative insult. The increase in LOOH production in both sham and denervated muscle treated with AACOCF_3_ after addition of AA suggests that AA release is the main regulatory step for LOOH production during eicosanoid metabolism.

Next, we used the permeabilized fiber protocol and treatment with either catalase or AACOCF_3_ to identify and compare the relative composition of hydroperoxide production rates in several models of neurogenic atrophy including an aging time-course, accelerated sarcopenia with neuromuscular degeneration (*Sod1*^***−/−***^ mice), and end-stage ALS motor neuron disease (SOD1^G93A^ mice). Catalase or AACOCF_3_ inhibited ~ 50–66% of hydroperoxides in sarcopenic aged mice (Fig. 3i). Age-related hydroperoxides are a mixture of LOOHs produced in the cPLA_2_ pathway and O_2_^−^/H_2_O_2_ likely produced by the ETC (Supplemental Fig. 2 Inset). In fibers from *Sod1*^***−/−***^, SOD1^G93A^, and denervated mice, the increased basal hydroperoxides are almost completely inhibited by the cPLA_2_ inhibitor AACOCF_3_ (Supplemental Fig. 2). AACOCF_3_ inhibits significantly more of the basal hydroperoxides in these models than catalase and in total inhibits ~ 80–90% of hydroperoxides (Fig. 3i). LOOHs produced by the cPLA_2_ pathway are induced in all tested models of neurogenic atrophy and are the primary component of basal hydroperoxides.

### H_2_O_2_ scavengers do not protect against denervation-induced muscle hydroperoxide production or atrophy

To more specifically test the contribution of increased H_2_O_2_ to neurogenic atrophy pathogenesis, we used two transgenic mouse models (skmMCAT and skmPRDX3) that overexpress mitochondrial H_2_O_2_ scavengers in skeletal muscle. The skmMCAT model expresses mitochondrially-targeted human catalase (mCAT) in muscle^28^. Catalase is an H_2_O_2_ scavenger normally localized to the peroxisome. The skmMCAT model uses a flox-stopped version of the construct to express catalase specifically in muscle^29^. The skmPRDX3 model over expresses peroxiredoxin 3 (PRDX3) in muscle also using a flox-stopped transgene. Peroxiredoxin 3 is an endogenous mitochondrial H_2_O_2_ scavenger^30^. Analysis of skmMCAT expression using Western blot, targeted mass-spectrometry, and qRT-PCR revealed a similar level of mCAT gene expression compared to mouse catalase and approximately a three–ninefold increase in total catalase content in the skeletal muscle of skmMCAT mice compared to controls (Supplemental Fig. 3a-d). mCAT expression did not change protein content of other antioxidant or metabolic enzymes measured by mass spectrometry (Supplemental Table 1). PRDX3 was overexpressed approximately two orders of magnitude in gastrocnemius muscles of the skmPRDX3 model (Supplemental Fig. 3e-f).

**Figure 4:**
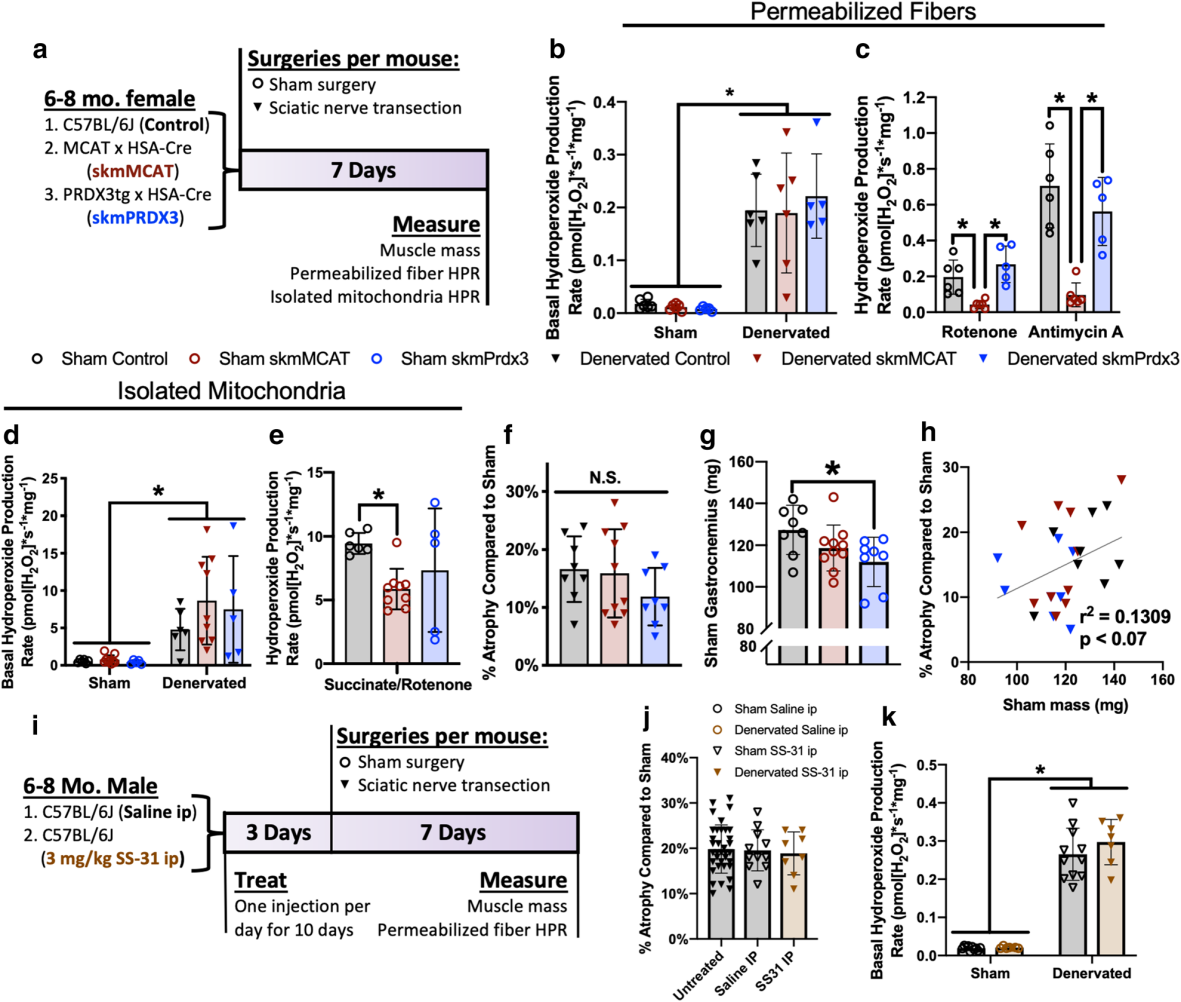
H_2_O_2_ scavengers do not protect against denervation-induced muscle hydroperoxide production or atrophy. (**a**) Experimental design. (**b**) Basal hydroperoxide production rate in sham and denervated permeabilized gastrocnemius fibers from control, skmMCAT, and skmPRDX3 mice. Statistical significance determined by ordinary two-way ANOVA with tukey’s post hoc test. (**c**) Hydroperoxide production rate after addition of ETC inhibitors in sham permeabilized gastrocnemius fibers from control, skmMCAT, and skmPRDX3 mice. Significantly different variation identified by Bartlett’s test. Statistical significance determined by Welch’s ANOVA test with Dunnett’s T3 post hoc test. (**d**) Basal hydroperoxide production rate in sham and denervated isolated gastrocnemius mitochondria from control, skmMCAT, and skmPRDX3 mice. Statistical significance determined by ordinary two-way ANOVA with tukey’s post hoc test. (**e**) Hydroperoxide production rate with the ETC inhibitor rotenone in sham isolated gastrocnemius mitochondria from control, skmMCAT, and skmPRDX3 mice. Significantly different variation identified by Bartlett’s test. Statistical significance determined by Welch’s ANOVA test with Dunnett’s T3 post hoc test. (**f**) Percent atrophy in denervated gastrocnemius relative to same animal sham of control and (**g**) sham gastrocnemius mass in control (n = 8), skmMCAT (n = 10), and skmPRDX3 (n = 8) mice. Statistical significance determined by ordinary one-way ANOVA with tukey’s post hoc test. (**h**) Correlation between sham gastrocnemius mass partially explains percent atrophy of denervated muscle in control (n = 8), skmMCAT (n = 10), and skmPRDX3 (n = 8) mice. Statistical significance determined by linear regression. All mice were female. (**i**) Experimental design for SS-31 injection experiment. (**j**) Percent atrophy in untreated (n = 35), saline-injected (n = 11), and SS-31-injected (n = 8) denervated gastrocnemius muscles compared to same animal sham. Statistical significance determined by ordinary one-way ANOVA with Tukey’s post hoc test. (**k**) Basal hydroperoxide production rates in saline-injected (n = 11) and SS-31-injected (n = 7) permeabilized fibers from sham and denervated gastrocnemius muscles. Statistical significance determined by ordinary two-way ANOVA with Tukey’s post hoc test. All mice were male. All plots represent mean ± standard deviation. *p < 0.05 for designated comparison.

To measure the impact of H_2_O_2_ generation on muscle atrophy induced by denervation, we performed sciatic nerve transection on control, skmMCAT, and skmPRDX3 mice and analyzed muscle mass and hydroperoxide production rates in isolated mitochondria and permeabilized fibers from gastrocnemius muscles after seven days (Fig. 4a). We find that sham muscle fibers and isolated mitochondria from skmMCAT but not skmPRDX3 mice have decreased hydroperoxide production rates after addition of ETC inhibitors compared to wildtype mice as expected for the increased H_2_O_2_ scavenging potential in muscle from these mice (Fig. 4c,e). However, neither skmMCAT or skmPRDX3 mice exhibit decreased peroxide production rates in denervated muscle fibers or isolated mitochondria compared to wildtype mice (Fig. 4b,d). Importantly, increased scavenging of H_2_O_2_ in muscle of skmMCAT and skmPRDX3 mice does not protect the mice from denervation induced loss of muscle mass (Fig. 4f). skmPRDX3 mice do tend to show a lower percent of atrophy in the denervated muscle compared to sham control, however this was not statistically significant. Interestingly, the skmPRDX3 mice have significantly smaller sham muscles, and a linear regression analysis shows that the size of the sham muscle partially explains variations in percent atrophy of the denervated gastrocnemius muscle compared to sham (Fig. 4g,h). Muscle fibers from skmMCAT mice and isolated muscle mitochondria efficiently scavenged H_2_O_2_ under control conditions, but increased H_2_O_2_ scavenging was not sufficient to protect against denervation-induced muscle hydroperoxide production or atrophy. Together our evidence suggests that increased muscle mitochondrial H_2_O_2_ production is not causal for muscle atrophy in response to denervation.

The tetrapeptide SS-31 was initially reported to have direct H_2_O_2_ antioxidant scavenging and to a lesser extent LOOH scavenging^25^. More recently, it has been suggested to exert its protective effects, including those in several age-related conditions, by virtue of its targeting to cardiolipin, with enhancement of ETC function, reduced ROS, and improved ATP production^31^. We treated wildtype mice for 10 days with daily injections of saline or SS-31 (3 mg/kg body weight). After 3 days of treatment, we performed sciatic nerve transection and analyzed muscle mass and hydroperoxide production 7 days later (Fig. 4i). SS-31 does not protect against muscle atrophy or increased basal hydroperoxide production in denervated muscle (Fig. 4j,k). In summary, the results of these three interventions suggest that H_2_O_2_ signaling is not required to induce muscle wasting, but instead denervation is primarily associated with increased LOOHs.

### Denervation induces cPLA_2_ activity and downstream eicosanoids in skeletal muscle resulting in LOOH production

**Figure 5:**
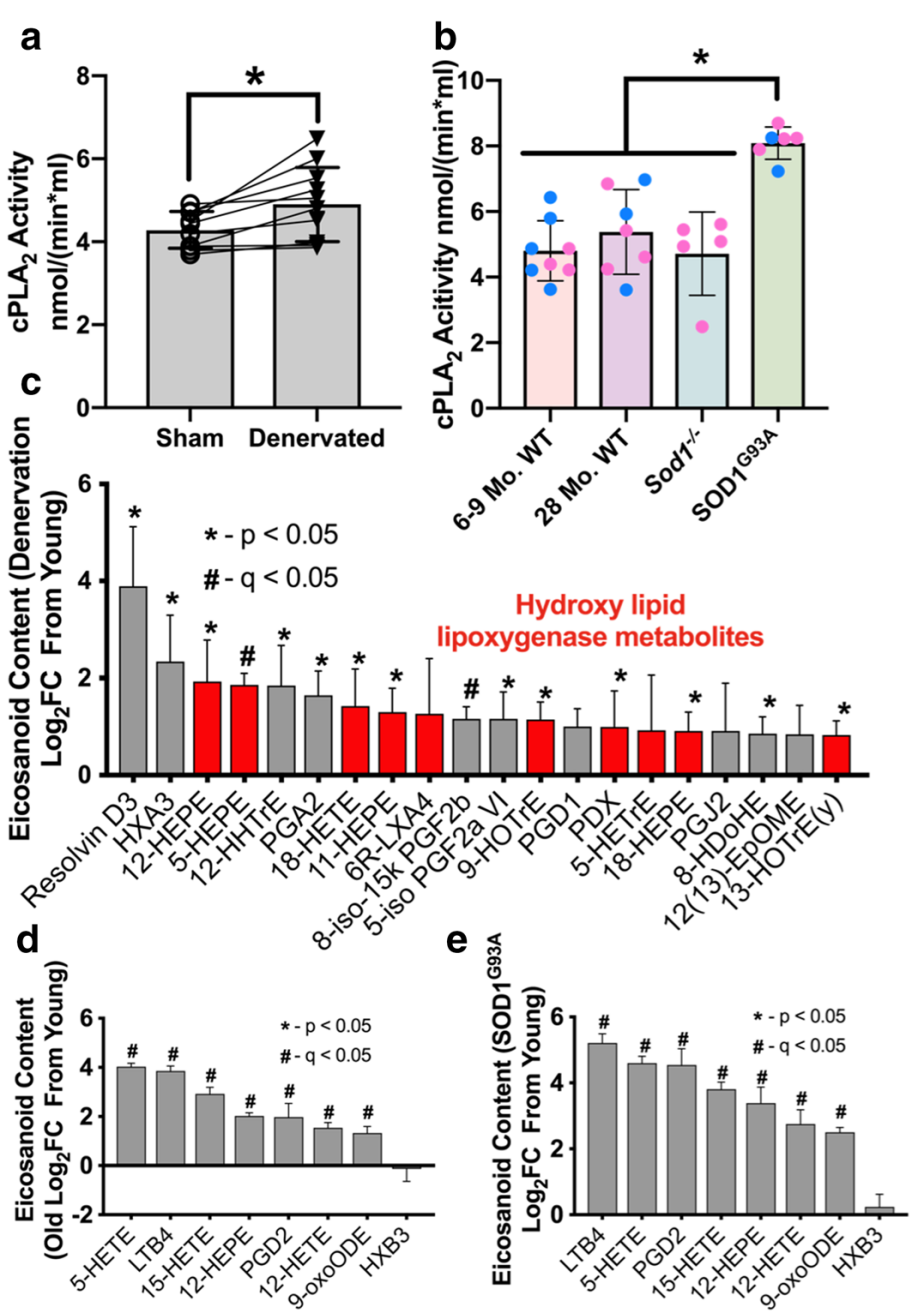
Loss of innervation induces cPLA_2_ activity and downstream eicosanoids in skeletal muscle. (**a**) cPLA_2_ activity in sham and denervated gastrocnemius muscles from male WT mice (n = 11 in triplicate). Statistical significance determined by two-tailed student's t-test. (**b**) cPLA_2_ activity in young (n = 8), old (n = 7), *Sod1*^***−/−***^ (n = 5), and SOD1^G93A^ (n = 6) gastrocnemius muscles from female (pink) and male (blue) mice performed in triplicate. Statistical significance determined by ordinary one-way ANOVA with Tukey’s post hoc test. *p < 0.05 for designated comparison. Eicosanoid content in gastrocnemius muscles expressed as log_x_ fold change from young control (n = 6) for (**c**) sciatic nerve transection (7 days denervation) (n = 4), (**d**) Old (28 month) wild-type (n = 4), and (**e**) end-stage SOD1^G93A^ mice (n = 4). All plots represent mean ± standard deviation. Statistical significance determined by two-tailed student’s t-test (*p < 0.05 compared to control) with Benjamini–Hochberg FDR correction (#q < 0.05 compared to control).

We previously reported an increase in cPLA_2_ protein content in denervated muscle compared to sham muscle^13^. In the current study, we measured cPLA_2_ activity in gastrocnemius muscles from sham and denervated mice seven days after sciatic nerve transection. cPLA_2_ activity is significantly elevated in denervated muscle fibers (Fig. 5a). We also measured cPLA_2_ activity in gastrocnemius muscles from young 6–9 month and old 28 month wild-type, onset *Sod1*^***−/−***^, and end-stage SOD1^G93A^ mice. cPLA_2_ activity was significantly elevated in wasting muscle from SOD1^G93A^ mice but not sarcopenic or *Sod1*^***−/−***^ mice (Fig. 5b).

To better understand the nature of the LOOH response following loss of innervation, we used a targeted lipidomics approach to quantify the content of eicosanoids in gastrocnemius muscle from young control and seven-day post-denervation mice. We find that multiple eicosanoids downstream of cPLA_2_ are increased in muscle from 7-day denervated mice (Fig. 5c). In the lipoxygenase pathway, phospholipid hydroperoxide glutathione peroxidase (GPX4) converts LOOH intermediates to hydroxylipids^18,20^. Of the increased eicosanoids in denervation, 9 out of 16 are hydroxy lipids found downstream of LOOH intermediates in the lipoxygenase pathway.

We chose a targeted panel of eight eicosanoids based on our results in denervated muscle and measured their content in muscle from aged and SOD1^G93A^ mice. Seven out of the eight measured eicosanoids were upregulated in aged and SOD1^G93A^ gastrocnemius compared to control (Fig. 5d,e). We found significant upregulation of eicosanoids in all neurogenic atrophy models compared to control.

### In vivo cPLA2_2_ inhibition protects against denervation-induced muscle hydroperoxide production and atrophy

**Figure 6:**
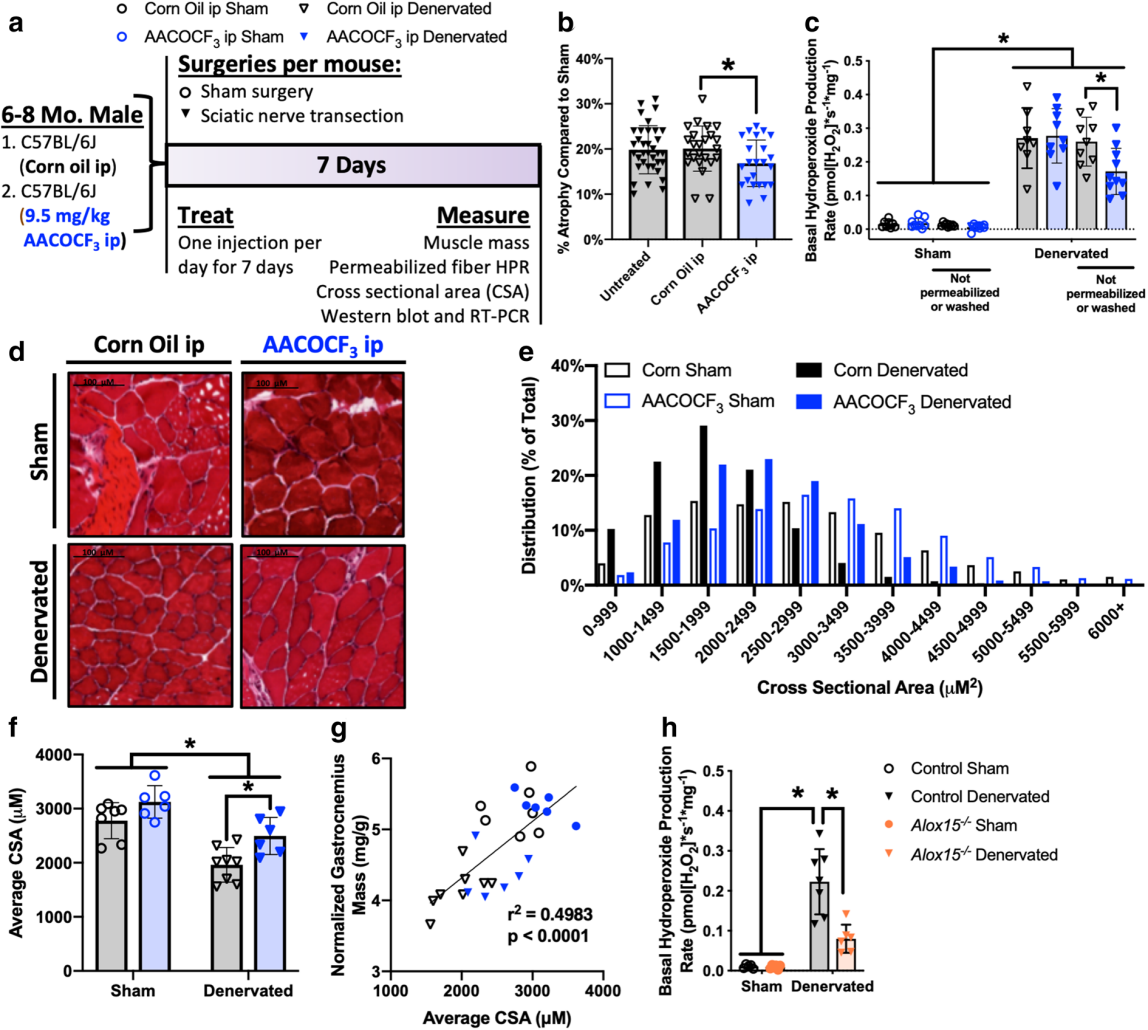
In vivo cPLA2_2_ Inhibition protects against denervation-induced muscle hydroperoxide production and atrophy. (**a**) Experimental design. (**b**) Percent atrophy of denervated gastrocnemius relative to sham of the same animal in male untreated (n = 35), corn oil-injected (n = 24), and AACOCF_3_ injected (n = 23) mice 7 days after sciatic nerve transection. Significance determined by two-tailed student’s t-test (vehicle versus AACOCF_3_). Untreated group shown as a comparison to vehicle treated. (**c**) Basal hydroperoxide production rate in sham and denervated gastrocnemius fibers from corn oil-injected (n = 8 permeabilized, n = 9 unpermeabilized), and AACOCF_3_ injected (n = 9 permeabilized, n = 10 unpermeabilized) mice 7 days after sciatic nerve transection. Significance determined by ordinary two-way ANOVA with Tukey’s post hoc test comparing within standard protocol or within not permeabilized or washed protocol. (**d**) Representative cross-sectional area (CSA) images using H&E stain and 20 × optical zoom (scale bar 100 μm). (**e**) Histogram of fiber CSA from corn oil injected (n = 7 sham and n = 8 denervated) and AACOCF_3_ injected (n = 6 sham and denervated) mouse gastrocnemius muscle. Statistical significance determined by Chi square. ~ 500 fibers were quantified per sample. (**f**) Quantification of average fiber CSA from corn oil injected (black, n = 7 sham and n = 8 denervated) and AACOCF_3_ injected (blue, n = 6 sham and denervated) mouse gastrocnemius muscle. Statistical significance determined by ordinary two-way ANOVA with Tukey’s post hoc test. (**g**) Correlation between normalized gastrocnemius mass and average gastrocnemius fiber CSA from corn oil injected (n = 7 sham and n = 8 denervated) and AACOCF_3_ injected (n = 6 sham and denervated) mice. Statistical significance determined by linear regression. All untreated, corn oil-injected, and AACOCF_3_-injected mice were male. (**h**) Basal hydroperoxide production rate in sham and denervated gastrocnemius fibers from female control (n = 7) or *Alox15*^***−/−***^ (n = 6) mice 7 days after sciatic nerve transection. Significance determined by ordinary two-way ANOVA with Tukey’s post hoc test. All plots represent mean ± standard deviation. *p < 0.05 for designated comparison.

To determine in vivo if increased production of LOOHs generated by cPLA_2_ plays a causal role in denervation atrophy, we performed sciatic nerve transection surgeries and treated mice with a daily ip injection of 9.5 mg/kg body weight AACOCF_3_ or corn oil vehicle control for 7 days then measured the effect of cPLA_2_ inhibition on muscle hydroperoxide production, atrophy, and individual fiber size (Fig. 6a). AACOCF_3_ is a potent cell-permeable inhibitor of cPLA_2_ and previous publications demonstrated this dose inhibited cPLA_2_ in vivo in spinal cords^32^. Vehicle injected mice show a similar amount of atrophy compared to untreated controls (~ 20% atrophy in denervated muscle compared to sham at 7 days) (Fig. 6b). AACOCF_3_-injected mice undergo significantly less atrophy (~ 17%) at 7 days, resulting in a rescue of 16% of atrophy (Fig. 6b). We have repeated this experiment in several cohorts of mice, and each cohort saw similar protection from atrophy (data not shown).

We measured basal hydroperoxide production in permeabilized fibers from sham and denervated gastrocnemius muscle with and without AACOCF_3_ treatment. The standard protocol for fiber preparation found no difference in basal hydroperoxide production with AACOCF_3_ treatment. However, the protection in atrophy in the treatment group suggested some drug effect. Because AACOCF_3_ is a reversible inhibitor, we hypothesized it may be washed out during the dilution steps in the permeabilization protocol. We performed mechanical separation of fibers from vehicle and treated mice and immediately measured hydroperoxide production rates omitting the permeabilization and washing steps. No difference was found in fibers from vehicle-injected mice with or without permeabilization, therefore this protocol is suitable for measuring basal hydroperoxide production (Fig. 6c). Using this fiber preparation protocol, we observe a significant reduction in basal hydroperoxides (~ 34%) in denervated fibers from AACOCF_3_-injected mice compared to control (Fig. 6c).

Denervation results in a significant decrease in average muscle fiber cross-sectional area (CSA) in gastrocnemius muscles from both vehicle- (29%) and AACOCF_3_-treated (20%) mice (Fig. 6d–f). However, we observe a significant treatment effect in response to AACOCF_3_, which is associated with an increase in individual denervated fiber CSA (27%) compared to vehicle treated mice (Fig. 6d–f). Denervated muscle contains an increased proportion of small muscle fibers compared to sham, but we observe that AACOCF_3_ treatment increases the proportion of large muscle fibers in both sham and denervated muscles and increases average CSA of denervated fibers compared to vehicle control (Fig. 6e–f). Average CSA area is linearly correlated with gastrocnemius mass, which suggests that denervation primarily induces muscle atrophy through individual fiber atrophy that is partially rescued with AACOCF_3_ treatment (Fig. 6g). We previously reported that mice lacking 12/15-lipoxygenase (*Alox15*^***−/−***^) were protected from denervation atrophy^33^. Here we report that the basal hydroperoxide production rate in *Alox15*^***−/−***^ denervated fibers is significantly decreased (~ 64%) (Fig. 6h). These experiments provide evidence that LOOHs produced by the cPLA_2_ pathway play a causal role in neurogenic muscle atrophy and are a viable target to protect against loss of muscle mass in neurogenic atrophy.

### AACOCF_3_ treatment does not decrease cPLA_2_ pathway protein content

**Figure 7:**
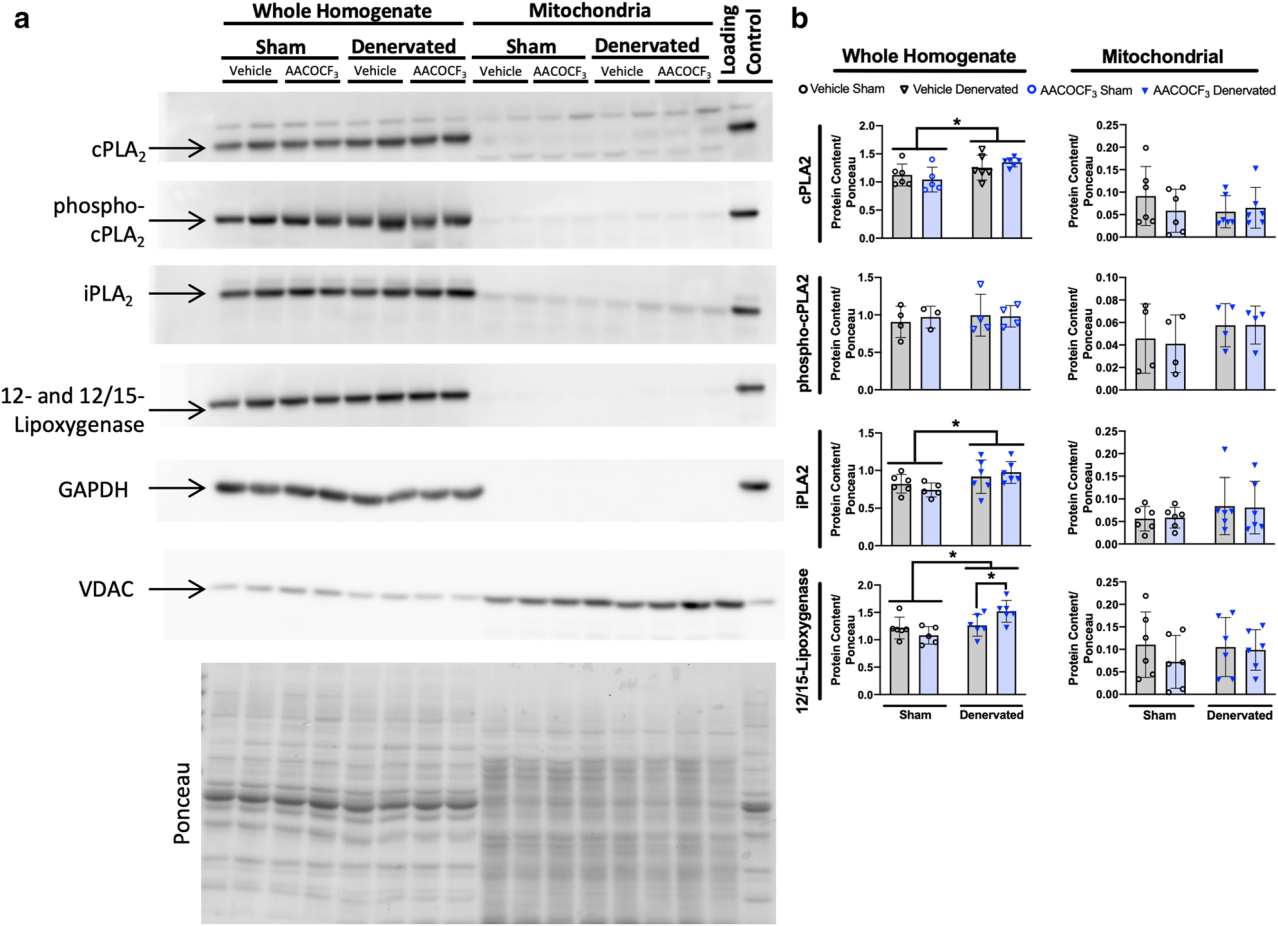
AACOCF_3_ treatment does not decrease cPLA_2_ pathway protein content. (**a**) Representative Western blot images and (**b**) quantifications in whole muscle homogenate and mitochondrial fraction of sham and denervated gastrocnemius muscles from male mice treated with 7 days of corn oil or AACOCF_3_ injections (n = 5–6). Ponceau images of each blot were taken, then the blots were cut to probe with individual primary antibodies. n = 2 samples were run per blot. Multiple blots were compared by loading a control sample on each blot and normalizing the probed bands to the loading control and then to the control sample. The blots were processed in parallel. Full blot images are available in Supplemental Fig. 4. Statistical significance determined by two-way ANOVA with Tukey’s post hoc test for each protein. All plots represent mean ± standard deviation. *p < 0.05 for designated comparison.

We next measured protein content of cPLA_2_, calcium-independent mitochondrial phospholipase A_2_ (iPLA_2_), and 12- and 12/15-lipoxygenase following denervation and cPLA_2_ inhibition in vivo. We measured protein content of key components of the cPLA_2_ pathway in both muscle whole homogenate and isolated mitochondria. Our analysis reveals that loss of innervation increases protein content of cPLA_2_, iPLA_2_, and 12- and 12/15-lipoxygenase in whole homogenate of the gastrocnemius muscle (Fig. 7a,b). In addition, AACOCF_3_ treatment significantly increases 12- and 12/15-lipoxygenase in denervated muscle compared to vehicle-treated denervated muscle (Fig. 7a,b). cPLA_2_ can be activated by increased intracellular calcium or phosphorylation by MAP kinase^34^. We observe no difference in phosphorylated cPLA_2_ content in whole homogenate. We also observe no difference in isolated mitochondrial content of cPLA_2_, phosphorylated cPLA_2_, iPLA_2_, or 12- and 12/15-lipoxygenase. Denervation does not increase activation of cPLA_2_ by phosphorylation (data not shown). Furthermore, AACOCF_3_ treatment inhibits cPLA_2_ activity but does not decrease expression or content of PLA_2_ pathway enzymes. These results suggest that denervation induces an increase in PLA_2_ pathway enzyme protein content, which results in the increased cPLA_2_ activity we observe in denervation.

### cPLA_2_ inhibition mitigates oxidative stress in denervation

**Figure 8:**
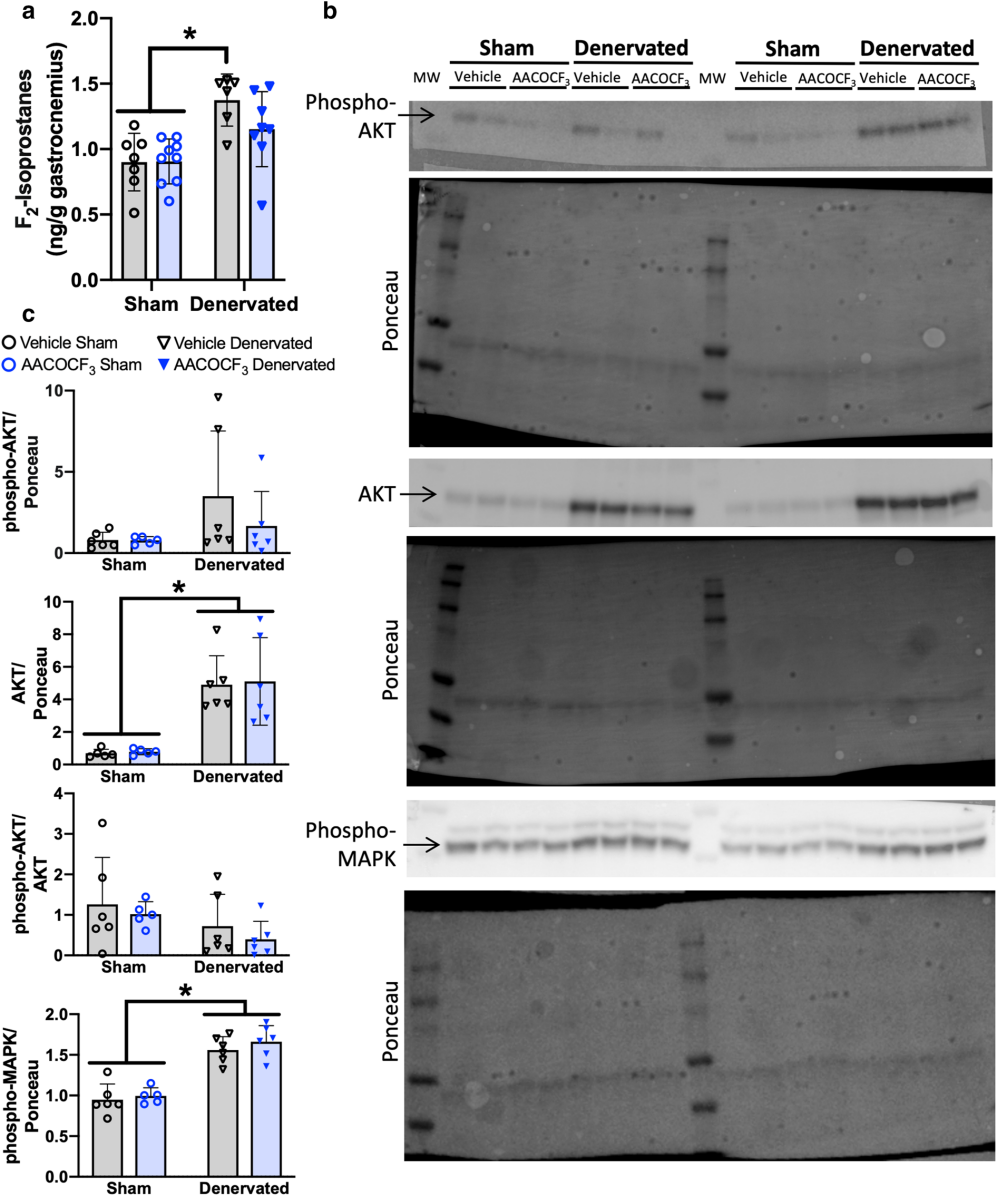
cPLA_2_ inhibition mitigates oxidative stress and eicosanoid signaling changes in denervation. We analyzed the mechanism of protection in sham or denervated gastrocnemius muscles from male mice treated for 7 days with vehicle or AACOCF_3_ ip injections. (**a**) The level of f_2_-Isoprostanes (vehicle n = 7; AACOCF_3_ n = 9). (**b**) IGF-1/Insulin pathway protein content and phosphorylation representative western blots and (**c**) Quantification (n = 6). Ponceau images of each blot were taken, then the blots were cut to probe with individual primary antibodies. All samples for each primary antibody probe were run on the same blot (n = 4 per blot). Full blot images are available in Supplemental Fig. 5. Statistical significance determined by two-way ANOVA with Tukey’s post hoc test. All plots represent mean ± standard deviation. *p < 0.05 for designated comparison. All mice were male.

We measured several parameters to determine if LOOH production in the cPLA_2_ pathway contributes to atrophy primarily through oxidative damage or eicosanoid signaling. F_2_-isoprostanes are a biomarker of oxidative stress formed by non-enzymatic reaction of AA with a free radical^35^. F_2_-isoprostane content is increased in denervated muscle, but treatment with AACOCF_3_ mitigates this increase (Fig. 8a). The cPLA_2_ knockout mouse were previously identified to have cardiac fiber hypertrophy via modulating IGF-1 pathway signaling^36^. We measured the content and phosphorylation of several proteins in the IGF-1 signaling pathway. Loss of innervation increases AKT protein content and MAP kinase phosphorylation (Fig. 8b,c). However, treatment with AACOCF_3_ did not affect these changes after loss of innervation.

## Discussion

In this work, we report a novel effect of lipid hydroperoxide generation in the initiation of skeletal muscle atrophy following loss of innervation. This is a new mechanism for induction of muscle atrophy by lipid mediators. Our studies further provide evidence for potential intervention and modulation of muscle atrophy by cPLA_2_ pathway inhibition that could be relevant for sarcopenia and other diseases of muscle wasting including ALS and nerve injury. cPLA_2_-derived LOOHs hold clinical relevance, as vastus lateralis biopsies from octogenarians also show LOOH production inhibited by AACOCF_3_^37^. These findings represent a paradigm shift in the understanding of ROS in muscle: previously research focused intensely on mitochondrial ETC-derived O_2_^−^, H_2_O_2_, and lipid peroxidation as causal factors in sarcopenia and not LOOHs produced in the cPLA_2_ pathway. Increased scavenging of muscle mitochondrial H_2_O_2_ did not protect against denervated muscle hydroperoxide production or atrophy, although whole body mCAT expression and SS-31 injection were previously found to protect aged muscle against H_2_O_2_ production or oxidative stress^28,38,39^. While we have focused on basal hydroperoxide production primarily composed of LOOHs, a recent report shows that activated mitochondria from aged skeletal muscle produce higher concentrations of superoxide (O_2_^−^) and H_2_O_2_ at physiological ADP concentrations, which may explain the mechanism of MCAT and SS-31 protection^40^. In the mitochondrial fraction, we observe cPLA_2_, iPLA_2_, and 12- and 12/15-lipoxygenase proteins, and elevated LOOH production in denervated muscles. We previously reported that both the cPLA_2_ inhibitor AACOCF_3_ and the glutathione peroxidase mimetic, ebselen, decreased basal hydroperoxide production in isolated mitochondria from denervated muscle to a greater extent than catalase^13^. Together, these results suggest some parts of the eicosanoid pathway are taking place in the mitochondrial fraction of denervated muscles^13^.

cPLA_2_ has been previously reported to be a negative regulator of cardiac and skeletal muscle hypertrophy^36^. Under physiological conditions, cPLA_2_ negatively regulates individual muscle fiber size by inhibiting IGF-1 signaling. cPLA_2_ releases AA from membranes, which is metabolized enzymatically in the cyclooxygenase, lipoxygenase, cytochrome p450, and anandamide pathways and non-enzymatically into isoprostanes and nitroalkenes^41^. The cyclooxygenase (COX) and lipoxygenase (LOX) pathways both produce LOOHs as intermediates^41^. cPLA_2_ and cyclooxygenase inhibitors are an ongoing topic of study for ALS treatment, although previous publications focus primarily on neuronal expression and inhibition^42,43^. The 12/15-Lipoxygenase is implicated in the pathology of a range of diseases^16^. We previously reported that *Alox15*^***−/−***^ mice were partially protected from muscle loss after sciatic nerve transection, and here we show they also have significantly decreased skeletal muscle LOOH production in response to denervation^33^. However, removing 12/15-lipoxygenase is not sufficient to completely prevent loss of muscle mass or hydroperoxide production. This suggests that multiple eicosanoid related enzymes are responsible for LOOH production in denervation and that there are multiple targetable steps in the pathway for intervention. While AACOCF_3_ is a more potent inhibitor of cPLA_2_ than iPLA_2_ or sPLA_2_, it also inhibits cyclooxygenase activity^44^. Production of LOOHs by cyclooxygenases could explain the total effectiveness of AACOCF_3_ ex vivo (~ 90%) compared to the partial effectiveness of 12/15-lipoxygenase knockout at inhibiting LOOH production (~ 64%)^44^. By identifying the other responsible enzymes and developing or testing inhibitors, we can more specifically inhibit LOOH production. LOOHs could cause atrophy through oxidative stress and signaling changes. Denervation increases F_2_-isoprostane content that is blunted with cPLA_2_ inhibition by AACOCF_3_. We previously reported denervation induces proteasome-mediated protein degradation^33^. However, deletion of 12/15-lipoxygenase protects against this induction in protein degradation, which suggests that cPLA_2_ regulates muscle size partially through downstream metabolites affecting proteostasis. Overall, we show that the cPLA_2_ pathway negatively regulates muscle mass. Enzymes in the cPLA_2_ pathway could be targeted using small molecule inhibitors to treat several atrophy conditions including aging, nerve injury, and ALS.

## Materials and methods

### Animals and animal models

All animal experiments were conducted in accordance with the guidelines for the care and use of laboratory animals of the University of Oklahoma Health Sciences Center (OUHSC) and Oklahoma Medical Research Foundation (OMRF). The study was approved by the Institutional Animal Care and Use Committees at the OUHSC and the OMRF. All experiments used mice from strains on a C57BL/6 J genetic background currently housed in the mouse colony of Dr. Van Remmen at the OMRF or Veterans Affairs (VA) Medical Center. Male 4 month old wild-type and 24 month old wild-type and *Nrf2*^***−/−***^ (B6.129X1*-Nfe2l2*^*tm1Ywk*^/J) gastrocnemius mass and basal hydroperoxide data was previously reported^22^. End-stage (135–152 days) SOD1^G93A^ mouse [B6-Tg(SOD1-G93A)1Gur/J(G93AGur1)] gastrocnemius mass and basal hydroperoxide data for vehicle and catalase or AACOCF_3_ treatment was previously reported^23^. *Sod1*^***−/−***^ mice were previously described^45^. mCAT-flox mice were previously described^29^. PRDX3tg-flox mice have a transgene that consists of CAG promoter, an LSL-STOP cassette flanked by loxP sites, and a human PRDX3 cDNA. In those mice, activation of Cre removes the LSL-STOP cassette, leading to expression of PRDX3. mCAT-flox or PRDX3tg-flox specifically target expression to muscle using a Cre-Lox system driven by human α-skeletal actin (HSA) directed Cre expression to make skmMCAT and skmPRDX3 lines respectively^46^. The transgenes contain a stop sequence at the start of the coding sequence flanked by LoxP sites. When the Cre is expressed in skeletal muscle fibers, the stop sequence is removed and the transgene is translated^47^. *Alox15*^***−/−***^ mice were acquired from The Jackson Laboratory (B6.129S2-*Alox15*^*tm1Fun*^/J) and were previously described^33^. Mice were caged in a pathogen free environment with free access to standard chow and water and maintained on a 12 h light/dark cycle. Mice were euthanized using a CO_2_ chamber and cervical dislocation. Hind limb muscles were dissected, weighed, and flash frozen in liquid nitrogen for biochemical analysis or used fresh for isolated mitochondria and permeabilized muscle fiber experiments. All experiments were carried out in the gastrocnemius muscle unless specified otherwise.

### Sciatic nerve transection surgeries and drug treatment regimens

Sciatic nerve transection and sham surgeries were performed as previously described on 6–8 month old C57BL/6 J, skmMCAT, skmPRDX3, and *Alox15*^***−/−***^ mice^11,13,33^. The mice were anesthetized in the induction chamber with isoflurane (2–3 units) then maintained under anesthesia using a mouse nose cone. The lateral thigh and buttock from the sciatic notch to the knee were shaved, and 100% ethanol and 2% chlorhexidine was used to clean the skin surface. A small incision was made from the sciatic notch to the knee (approximately 1 cm), and the sciatic nerve was exposed. The nerve was cut and a 5 mm piece removed. The ends of the nerve were folded back and sutured to prevent nerve regrowth. On the other leg, the nerve was exposed but not cut and removed. This limb served as a sham-operated control. The incision on each leg was closed using the PDS suture 5–0 and skin glue, and the mouse was placed on a warm heating pad until recovered. The mice were treated with 9.5 mg/kg ketaprofen dissolved in saline for three days following surgery to minimize pain. The mice were euthanized and dissected at 0.5, 1, 2, 4, 7, or 14 days after the surgery.

A cohort of male control mice were treated with arachidonyl trifluoromethyl ketone (AACOCF_3_; Enzo BML-ST335-0,050) or vehicle (corn oil) following sciatic nerve transection surgery. AACOCF_3_ was dissolved in DMSO (25 mg/ml) and then diluted in corn oil to 4 mM. Mice were treated with intraperitoneal (ip) injections of vehicle control (corn oil) or 9.5 mg/kg AACOCF_3_ once daily beginning with the first injection at the time of surgery and continuing until euthanasia seven days after surgery. Another cohort of mice was treated with SS-31 or vehicle control. Mice were treated daily with ip injections of 3 mg/kg SS-31 or vehicle (saline) control based on previous publications demonstrating efficacy of this dose in mouse skeletal muscle^38,48^. Sciatic nerve transection and sham surgeries were performed on the fourth day of SS-31 or vehicle injection treatment and mice were euthanized seven days later.

### Muscle fiber permeabilization and hydroperoxide production rate measurement

Preparation of permeabilized muscle fiber bundles with saponin and measurement of hydroperoxide production with Amplex UltraRed using the Oroboros Oxygraph-2k (O2k, OROBOROS Instruments, Innsbruck, Austria) with fluorometer were performed as previously described^22,23^. Amplex UltraRed measures hydroperoxide production (both hydrogen peroxide and lipid hydroperoxides). Horseradish peroxidase catalyzes a reaction between a hydroperoxide group and Amplex UltraRed to produce the fluorescent compound resorufin. The rate of resorufin production is detected as the change in fluorescence intensity. We performed a preliminary experiment adding increasing concentrations of GPX1 to a denervated permeabilized fiber and found 10 U/ml GPX1 more efficiently inhibited hydroperoxide production than lower or higher doses (data not shown). In Fig. 3c, the denervated gastrocnemius was removed from each animal then separated into four different permeabilized fiber bundles per animal. These bundles were untreated, treated with catalase, treated with GPX1, or treated with catalase and GPX1, respectively. Separate animals were used for the experiment in Fig. 3d. From the sham surgery leg, one control permeabilized gastrocnemius fiber bundle was prepared. From the denervated leg, three permeabilized gastrocnemius fiber bundles were prepared per animal. These were untreated (vehicle), treated with catalase, or treated with AACOCF_3,_ respectively. Permeabilized fibers were treated with 2000 U/ml catalase or 10 U/ml GPX1 during 30 min saponin permeabilization, 3 × 5 min wash steps, and in the O2k chamber during measurement. Permeabilized fibers were treated with 20 µM arachidonyl trifluoromethyl ketone (AACOCF_3_; Cayman Chemicals, Ann Arbor, MI) during 30 min saponin permeabilization and 3 × 5 min wash steps, but not in the O2k chamber during measurement. The addition of substrates and inhibitors during measurements was performed as previously described^22,23^. A dose response of Amplex UltraRed/resorufin fluorescence to H_2_O_2_ and lipid hydroperoxide (13(S)-HpODE and 15(S)-HpETE) concentrations was determined with and without the addition of substrates and inhibitors at the specified concentrations for H_2_O_2_ and 15(S)-HpETE (Fig. 3b,c). The supraphysiological catalase concentration increases background auto-oxidation of Amplex UltraRed, which increases background fluorescence rate. Auto-oxidation is accounted for in all samples by subtracting its rate for experimental measurements, however this induces increased variability in measurements with catalase. The actual inhibition of basal hydroperoxides by catalase in each model may be lower than that presented here.

### Mitochondrial isolation and hydroperoxide production rate

Mitochondria were isolated using the Chappell–Perry method, and hydroperoxide production rate was measured using Amplex Red fluorescent rate measurements in a plate-based fluorometer as previously described in gastrocnemius muscles^11,13^.

### Cytosolic phospholipase A_2_ (cPLA_2_) activity assay

cPLA_2_ activity was assessed using the Abcam Cytosolic Phospholipase A_2_ Assay Kit (ab133090) according to the manufacturer's instructions. 50 mg of gastrocnemius muscle was homogenized on ice in a glass homogenizer (size 21) in 500 μl homogenization buffer (50 mM HEPES, 1 mM EDTA, pH 7.4). The homogenate was centrifuged at 10,000 × *g* for 15 min at 4 °C. The supernatant was removed and filtered using Amicon 30 kDA filter (UFC803024) at 4,000 × *g* for 20 min at 4 °C. Protein concentration was determined using the Bradford assay. Samples were incubated with bromoenol lactone for 15 min at RT to inhibit iPLA_2_. 100 μg protein was loaded in triplicate for each sample. The assay was initiated with substrate solution and incubated for 60 min at RT. Enzyme catalysis with ended with DTNB/EGTA and absorbance was read at 414 nm and converted to enzyme activity using the manufacturer’s calculations.

### Eicosanoid measurements by targeted lipidomics

Snap frozen gastrocnemius from 4 to 6 month old young control, 4–6 month old 7 day denervated, 28 month old aged, and end-stage SOD1^G93A^ male mice were sent to the LIPID MAPS Lipidomics Core, https://www.ucsd-lipidmaps.org. at the University of California, San Diego. Eicosanoids were analyzed by LC–MS as previously described^49^. The data were analyzed using a Python 3 script. The script used the Bartlett variance test to analyze for unequal variance of each eicosanoid between control and experimental group. If variance was equal, each eicosanoid was compared with two-tailed student’s t-test. If variance was unequal, each eicosanoid was compared with Welch’s t-test. Benjamini–Hochberg FDR correction (q < 0.05) was used to determine statistical significance compared to control. Principal component analysis (PCA) plot were generated using ClustVis with default settings^50^.

### Individual muscle fiber cross sectional area

Fiber size was measured as previously described^51^. Gastrocnemius muscle was sectioned during sacrifice, mounted in optimum cutting temperature compound (OCT), and flash frozen in liquid nitrogen-cooled isopentane. Sections (10 μm) were mounted and stained with hematoxylin and then with Aeosin using a Leica ST5020 Multistainer Leica Microsystems, Wetzlar, Germany). Images were visualized and captured with Zeiss Axiovert 200 M microscope, Zeiss Axiocam MRC camera, and Zeiss AxioVision software V4.8.2.0 (Carl Zeiss AG, Oberkochen, Germany) at 10 × magnification. Cross-sectional area (CSA) was measured using ImageJ software for approximately 500 fibers per sample^52^.

### Western blot analysis

Western blots were performed as previously described using standard techniques^23^. Imaging was performed with G:BOX imaging system (Syngene) and quantified using GeneTools software (Syngene). Information on antibodies is available in Supplemental Table 2.

### Quantitative real-time polymerase chain reaction (RT-PCR)

RT-PCR was performed as previously described^53^. Total RNA was extracted from gastrocnemius using TRIzol reagent (Invitrogen, Carlsbad, CA, United States). Equal amounts of extracted RNA (1 μg) were converted to first strand cDNA using a cDNA synthesis kit (Bio-Rad, Herculus, CA, United States). Human catalase and mouse catalase mRNA levels were measured using Assay on Demand Hs00156308_m1 and Mm00437992_m1 (Applied Biosystems) as previously described^28^. RT-PCR was performed in Quant Studio 6 (Applied Biosystems, Foster City, CA, United States). The ΔΔC_t_ method was used to calculate relative mRNA expression.

### Lipid peroxidation by F_2_-isoprostanes

Levels of F_2_-isoprostanes in 100–150 mg of sham and denervated gastrocnemius muscles after seven days of denervation and treatment with vehicle or AACOCF_3_ were determined by gas chromatography–mass spectrometry as previously described^22,54^. The level of F_2_-isoprostanes in muscle tissues was expressed as nanograms of 8-Iso-PGF_2α_ per gram of muscle mass.

### Quantification of protein abundance using amino acid sequence

We used targeted quantitative mass spectrometry to measure protein abundance of metabolic and antioxidant proteins in control and skmMCAT gastrocnemius muscles as previously described^22,23,55^. Skyline was used to monitor and process data from each peptide^56^.

### RNA extraction, library construction, and RNA sequencing (RNAseq)

Using TRIzol reagent (Invitrogen, CA, USA), total RNA was extracted from sham and denervated gastrocnemius that were collected at 0.5, 1, 2, 4, 7, and 14 days after sciatic nerve transection. The RNA integrity and concentration were first assessed using an Agilent Bioanalyzer (Agilent Technologies, Santa Clara, CA) to meet the experimental standard. The RNA samples were given to OMRF Clinical Genomics Center. Libraries were prepared using the TruSeq Stranded mRNA Library Kit (Illumina). The libraries were then sequenced on an Illumina NextSeq 500 to produce paired-end 75 base pair reads. The data collected were given to Discovery Bioinformatics Core at the Oklahoma Nathan Shock Center of Excellence in the Biology of Aging for data analysis.

RNA-seq data processing followed the guidelines and practices of the ENCODE and modENCODE consortia regarding proper experimental replication, sequencing depth, data and metadata reporting, and data quality assessment (https://www.encodeproject.org/documents/cede0cbe-d324-4ce7-ace4-f0c3eddf5972/) as previously described^57^. Raw sequencing reads (in a FASTQ format) were trimmed of residual adaptor sequences using Scythe software. Low quality bases at the beginning or the end of sequencing reads were removed using sickle then the quality of remaining reads was confirmed with FastQC. Further processing of quality sequencing reads was performed with utilities provided by the Tuxedo Suite software. Reads were aligned to the *Mus musculus* genome reference (GRCm38/mm10) using the TopHat component, then cuffquant and cuffdiff were utilized for gene-level read counting and differentially expression analysis. A false discovery rate threshold of 0.05 was used as selection criteria for differentially expressed genes between pairs of time points. Functional analysis to find overrepresented functional sets (GO, KEGG pathways) was performed using specialized R Bioconductor packages. Ingenuity Pathway Analysis (IPA, QIAGEN, Redwood City CA, https://www.qiagenbioinformatics.com/products/ingenuitypathway-analysis) was used to explore significant gene networks and pathways interactively.

Promoter motif analysis in differentially expressed genes was performed using Hypergeometric Optimization of Motif EnRichment (HOMER) version 4.9.1^58^. Motifs enrichment was conducted in gene’s proximal promoter (400 bp upstream and 100 upstream). Venn diagram was drawn in Microsoft Powerpoint. Venn diagram was drawn in Microsoft Powerpoint.

### Statistical analyses

The results were analyzed using Microsoft Office Excel and GraphPad Prism 7.0b for Mac OS X (GraphPad Software, La Jolla, CA) unless otherwise specified. Statistical tests were determined according to experimental design as described in figure legends. Significantly different variation between groups was identified by Bartlett’s test. For pairwise comparisons, unpaired two-tailed t-test with *p* < 0.05 were used to determine significance for pairs with equal variance. For multiple groups, ordinary one-way ANOVA or ordinary two-way ANOVA and appropriate post hoc test with adjusted *p* < 0.05 determined significance for comparisons with equal variance. Statistical significance was determined by Welch’s ANOVA test with Dunnett’s T3 post hoc test for groups with unequal variance. Linear regression was used for correlation analysis.

## Supplementary information

Supplementary file1

Supplementary file2

Supplementary file3

Supplementary file4

## References

[1] Rizzoli R (2013). Quality of life in sarcopenia and frailty. Calcif. Tissue Int..

[2] Wickham C, Cooper C, Margetts BM, Barker DJ (1989). Muscle strength, activity, housing and the risk of falls in elderly people. Age Ageing.

[3] Janssen I, Shepard DS, Katzmarzyk PT, Roubenoff R (2004). The healthcare costs of sarcopenia in the United States. J. Am. Geriatr. Soc..

[4] Campbell MJ, McComas AJ, Petito F (1973). Physiological changes in ageing muscles. J. Neurol. Neurosurg. Psychiatry.

[5] Delbono O (2003). Neural control of aging skeletal muscle. Aging Cell.

[6] Rowan SL (2012). Denervation causes fiber atrophy and myosin heavy chain co-expression in senescent skeletal muscle. PLoS ONE.

[7] Brooks SV, Faulkner JA (1988). Contractile properties of skeletal muscles from young, adult and aged mice. J. Physiol..

[8] Wolf NS (2010). The Comparative Biology of Aging.

[9] Jang YC (2012). Dietary restriction attenuates age-associated muscle atrophy by lowering oxidative stress in mice even in complete absence of CuZnSOD. Aging Cell.

[10] Jang YC (2010). Increased superoxide in vivo accelerates age-associated muscle atrophy through mitochondrial dysfunction and neuromuscular junction degeneration. FASEB J..

[11] Muller FL (2007). Denervation-induced skeletal muscle atrophy is associated with increased mitochondrial ROS production. Am. J. Physiol. Regul. Integr. Comp. Physiol..

[12] Sakellariou GK (2014). Neuron-specific expression of CuZnSOD prevents the loss of muscle mass and function that occurs in homozygous CuZnSOD-knockout mice. FASEB J..

[13] Bhattacharya A (2009). Denervation induces cytosolic phospholipase A2-mediated fatty acid hydroperoxide generation by muscle mitochondria. J. Biol. Chem..

[14] Pollock N, Staunton CA, Vasilaki A, McArdle A, Jackson MJ (2017). Denervated muscle fibers induce mitochondrial peroxide generation in neighboring innervated fibers: role in muscle aging. Free Radic. Biol. Med..

[15] Adams CM, Ebert SM, Dyle MC (2017). Role of ATF4 in skeletal muscle atrophy. Curr. Opin. Clin. Nutr. Metab. Care.

[16] Singh NK, Rao GN (2019). Emerging role of 12/15-lipoxygenase (ALOX15) in human pathologies. Prog. Lipid Res..

[17] Street IP (1993). Slow- and tight-binding inhibitors of the 85-kDa human phospholipase A2. Biochemistry.

[18] Katsuki H, Okuda S (1995). Arachidonic acid as a neurotoxic and neurotrophic substance. Prog. Neurobiol..

[19] Higdon A, Diers AR, Oh JY, Landar A, Darley-Usmar VM (2012). Cell signalling by reactive lipid species: new concepts and molecular mechanisms. Biochem. J..

[20] Savaskan NE, Ufer C, Kuhn H, Borchert A (2007). Molecular biology of glutathione peroxidase 4: from genomic structure to developmental expression and neural function. Biol. Chem..

[21] Jakoby WB (1980). Enzymatic Basis of Detoxication.

[22] Ahn B (2018). Nrf2 deficiency exacerbates age-related contractile dysfunction and loss of skeletal muscle mass. Redox Biol..

[23] Pharaoh G (2019). Metabolic and stress response changes precede disease onset in the spinal cord of mutant SOD1 ALS mice. Front. Neurosci..

[24] Wu G, Fang YZ, Yang S, Lupton JR, Turner ND (2004). Glutathione metabolism and its implications for health. J. Nutr..

[25] Szeto HH (2006). Mitochondria-targeted peptide antioxidants: novel neuroprotective agents. AAPS J..

[26] Szeto HH (2006). Cell-permeable, mitochondrial-targeted, peptide antioxidants. AAPS J..

[27] Zilka O (2017). On the mechanism of cytoprotection by ferrostatin-1 and liproxstatin-1 and the role of lipid peroxidation in ferroptotic cell death. ACS Cent. Sci..

[28] Schriner SE (2005). Extension of murine life span by overexpression of catalase targeted to mitochondria. Science.

[29] Dai DF (2011). Mitochondrial oxidative stress mediates angiotensin II-induced cardiac hypertrophy and Galphaq overexpression-induced heart failure. Circ. Res..

[30] Chen L (2008). Reduction of mitochondrial H2O2 by overexpressing peroxiredoxin 3 improves glucose tolerance in mice. Aging Cell.

[31] Szeto HH (2014). First-in-class cardiolipin-protective compound as a therapeutic agent to restore mitochondrial bioenergetics. Br. J. Pharmacol..

[32] Liu NK (2014). Cytosolic phospholipase A2 protein as a novel therapeutic target for spinal cord injury. Ann. Neurol..

[33] Bhattacharya A (2014). Genetic ablation of 12/15-lipoxygenase but not 5-lipoxygenase protects against denervation-induced muscle atrophy. Free Radic. Biol. Med..

[34] Hefner Y (2000). Serine 727 phosphorylation and activation of cytosolic phospholipase A2 by MNK1-related protein kinases. J. Biol. Chem..

[35] Milne GL, Musiek ES, Morrow JD (2005). F2-isoprostanes as markers of oxidative stress in vivo: an overview. Biomarkers.

[36] Haq S (2003). Deletion of cytosolic phospholipase A2 promotes striated muscle growth. Nat. Med..

[37] Spendiff S (2016). Denervation drives mitochondrial dysfunction in skeletal muscle of octogenarians. J. Physiol..

[38] Siegel MP (2013). Mitochondrial-targeted peptide rapidly improves mitochondrial energetics and skeletal muscle performance in aged mice. Aging Cell.

[39] Umanskaya A (2014). Genetically enhancing mitochondrial antioxidant activity improves muscle function in aging. Proc. Natl. Acad. Sci. USA.

[40] Holloway GP (2018). Age-associated impairments in mitochondrial ADP sensitivity contribute to redox stress in senescent human skeletal muscle. Cell Rep..

[41] Hanna VS, Hafez EAA (2018). Synopsis of arachidonic acid metabolism: a review. J. Adv. Res..

[42] Kiaei M (2005). Integrative role of cPLA with COX-2 and the effect of non-steriodal anti-inflammatory drugs in a transgenic mouse model of amyotrophic lateral sclerosis. J. Neurochem..

[43] Solomonov Y, Hadad N, Levy R (2016). Reduction of cytosolic phospholipase A2alpha upregulation delays the onset of symptoms in SOD1G93A mouse model of amyotrophic lateral sclerosis. J. Neuroinflammation.

[44] Farooqui AA, Ong WY, Horrocks LA (2006). Inhibitors of brain phospholipase A2 activity: their neuropharmacological effects and therapeutic importance for the treatment of neurologic disorders. Pharmacol. Rev..

[45] Huang TT (1997). Superoxide-mediated cytotoxicity in superoxide dismutase-deficient fetal fibroblasts. Arch. Biochem. Biophys..

[46] Zhang Y (2013). CuZnSOD gene deletion targeted to skeletal muscle leads to loss of contractile force but does not cause muscle atrophy in adult mice. FASEB J..

[47] Lakso M (1992). Targeted oncogene activation by site-specific recombination in transgenic mice. Proc. Natl. Acad. Sci. USA.

[48] Campbell MD (2019). Improving mitochondrial function with SS-31 reverses age-related redox stress and improves exercise tolerance in aged mice. Free Radic. Biol. Med..

[49] Quehenberger O (2010). Lipidomics reveals a remarkable diversity of lipids in human plasma. J. Lipid Res..

[50] Metsalu T, Vilo J (2015). ClustVis: a web tool for visualizing clustering of multivariate data using principal component analysis and heatmap. Nucl. Acids Res..

[51] Brown LA (2015). Diet-induced obesity alters anabolic signalling in mice at the onset of skeletal muscle regeneration. Acta Physiol. (Oxf.).

[52] Rueden CT (2017). Image J2: ImageJ for the next generation of scientific image data. BMC Bioinform..

[53] Sataranatarajan K (2015). Neuron specific reduction in CuZnSOD is not sufficient to initiate a full sarcopenia phenotype. Redox Biol..

[54] Roberts LJ, Morrow JD (2000). Measurement of F(2)-isoprostanes as an index of oxidative stress in vivo. Free Radic. Biol. Med..

[55] Kinter CS (2012). A quantitative proteomic profile of the Nrf2-mediated antioxidant response of macrophages to oxidized LDL determined by multiplexed selected reaction monitoring. PLoS ONE.

[56] MacLean B (2010). Skyline: an open source document editor for creating and analyzing targeted proteomics experiments. Bioinformatics.

[57] Sataranatarajan K (2020). Molecular changes in transcription and metabolic pathways underlying muscle atrophy in the CuZnSOD null mouse model of sarcopenia. Geroscience.

[58] Heinz S (2010). Simple combinations of lineage-determining transcription factors prime cis-regulatory elements required for macrophage and B cell identities. Mol Cell.

