# Supplementary material for: Targeting cPLA2 derived lipid hydroperoxides as a potential intervention for sarcopenia
Source: Sci Rep. 2020 Aug 18;10:13968. doi: 10.1038/s41598-020-70792-7 (PMC7435184; doi:10.1038/s41598-020-70792-7)
Supplement: Supplementary file 1 — Supplementary file1 [file 41598_2020_70792_MOESM1_ESM.pdf]

TITLE

TARGETING CPLA$_2$ DERIVED LIPID HYDROPEROXIDES AS A POTENTIAL INTERVENTION FOR SARCOPENIA

AUTHORS

Gavin Pharaoh[1,2], Jacob L. Brown[2], Kavithalakshmi Sataranatarajan[2], Parker Kneis[2], Jan Bian[2], Rojina Ranjit[2], Niran Hadad[3,4], Constantin Georgescu[5], Peter Rabinovitch[6], Qitao Ran[7,8], Jonathan D. Wren[5], Willard Freeman[1,3,4,5], Michael Kinter[2], Arlan Richardson[3], Holly Van Remmen[1,2,4,9]*

[1] Physiology Department, University of Oklahoma Health Sciences Center, Oklahoma City, OK, USA

[2] Aging & Metabolism Research Program, Oklahoma Medical Research Foundation, Oklahoma City, OK, USA

[3] Reynolds Oklahoma Center on Aging, University of Oklahoma Health Sciences Center, Oklahoma City, OK, USA

[4] Oklahoma Center for Neuroscience, University of Oklahoma Health Sciences Center, Oklahoma City, OK, USA

[5] Genes & Human Disease Research Program, Oklahoma Medical Research Foundation, Oklahoma City, OK, USA

[6] Department of Pathology, University of Washington, Seattle, WA, USA

[7] Department of Cell Systems & Anatomy, UT Health San Antonio, TX, USA

[8]'South Texas Veterans Health Care System, San Antonio, TX, USA

[9] Oklahoma City VA Medical Center, Oklahoma City, OK, USA

*Corresponding Author: Holly Van Remmen, Ph.D.

Holly-VanRemmen@omrf.org

Lab Phone: (405)-271-2653

Office Phone: (405)-271-2520

Fax: (405) 271-3470

SUPPLEMENTARY MATERIAL

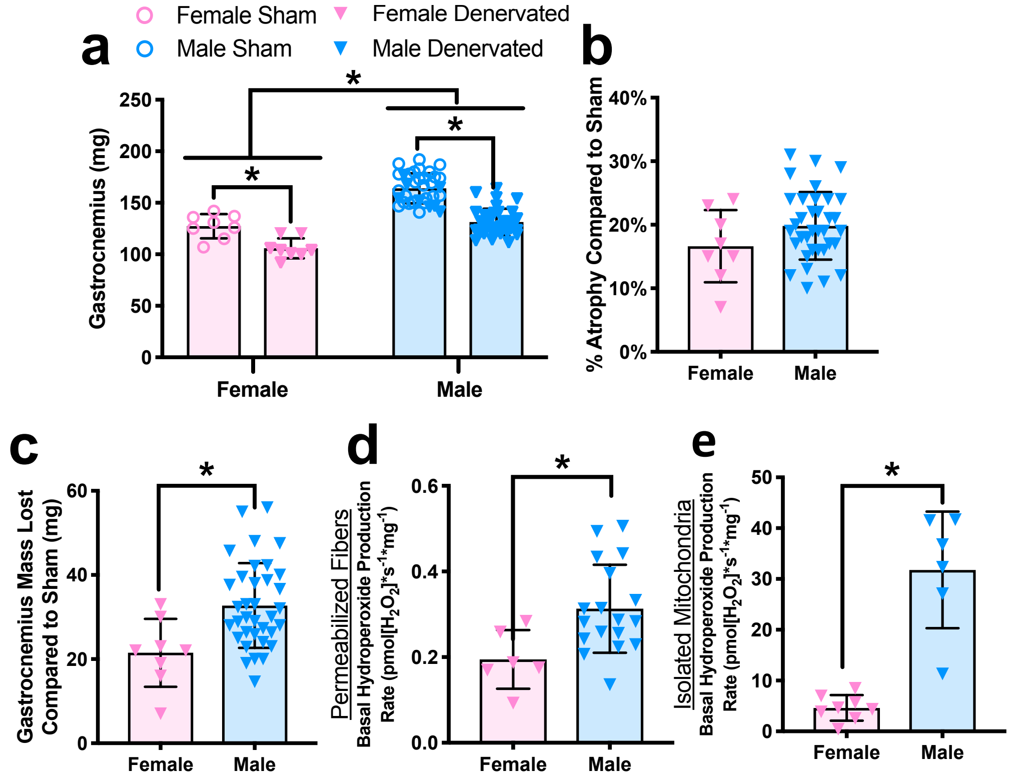

Supplemental Figure 1. Denervation Induces More Hydroperoxide Production and Muscle Atrophy in Male Mice. Response to denervation of male and female mice 7 days after sciatic nerve transection. **a)** Raw mass of sham and denervated gastrocnemius muscle (n = 8F and 35M). Statistical significance determined by ordinary two-way ANOVA with Tukey's post hoc test (*p<0.05 for marked comparison). **b)** Percent atrophy of denervated muscle relative to sham (n = 6F and 18M) and **c)** total gastrocnemius mass lost in the denervated muscle compared to sham in the same animal (n = 8F and 35M). **d)** Denervated permeabilized fiber basal hydroperoxide production rate (n = 6F and 18M) and **e)** denervated isolated mitochondria basal hydroperoxide production rate (n = 8F and 6M). Statistical significance determined by two-tailed student's t-test (*p<0.05 male versus female). Plots represent mean ± standard deviation.

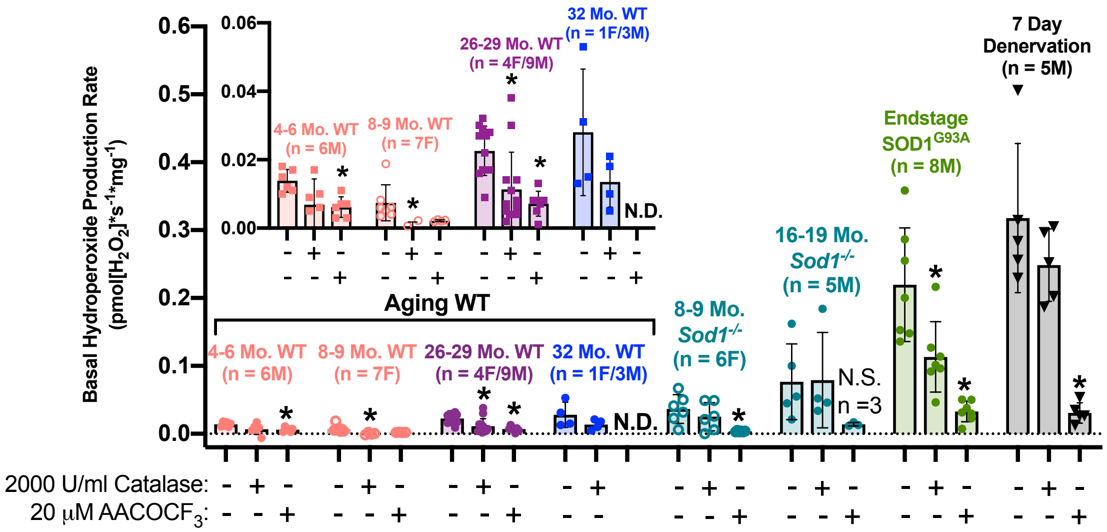

Supplemental Figure 2. cPLA2 Produces 50-90 Percent of Basal Hydroperoxides in Several Models of Neurogenic Atrophy. Basal hydroperoxide production rate in permeabilized gastrocnemius fibers of neurogenic atrophy models with 2000 U/ml catalase or 20 μM $AACOCF_3$ treatment. Inset, zoomed in changes in aging WT muscle. Statistical significance determined by ordinary one-way ANOVA with Tukey's post hoc test. All plots represent mean ± standard deviation (*p<0.05 versus vehicle). For 16-19 Mo. *Sod1*$^{-/-}$, n = 5M total, except catalase inhibition was only performed on 4 samples and $AACOCF_3$ inhibition was only performed on 3 samples due to limitations in the number of Oroboros O2k chambers available at the time of experimentation and thus was not powered to reach statistical significance. However, similar inhibition with $AACOCF_3$ was observed compared to 8-9 month old *Sod1*$^{-/-}$. End-stage SOD1^{G93A} basal hydroperoxide production rate and inhibition by catalase and $AACOCF_3$ was previously reported and included here for further comparison between multiple models[1]. N.D. – No data.

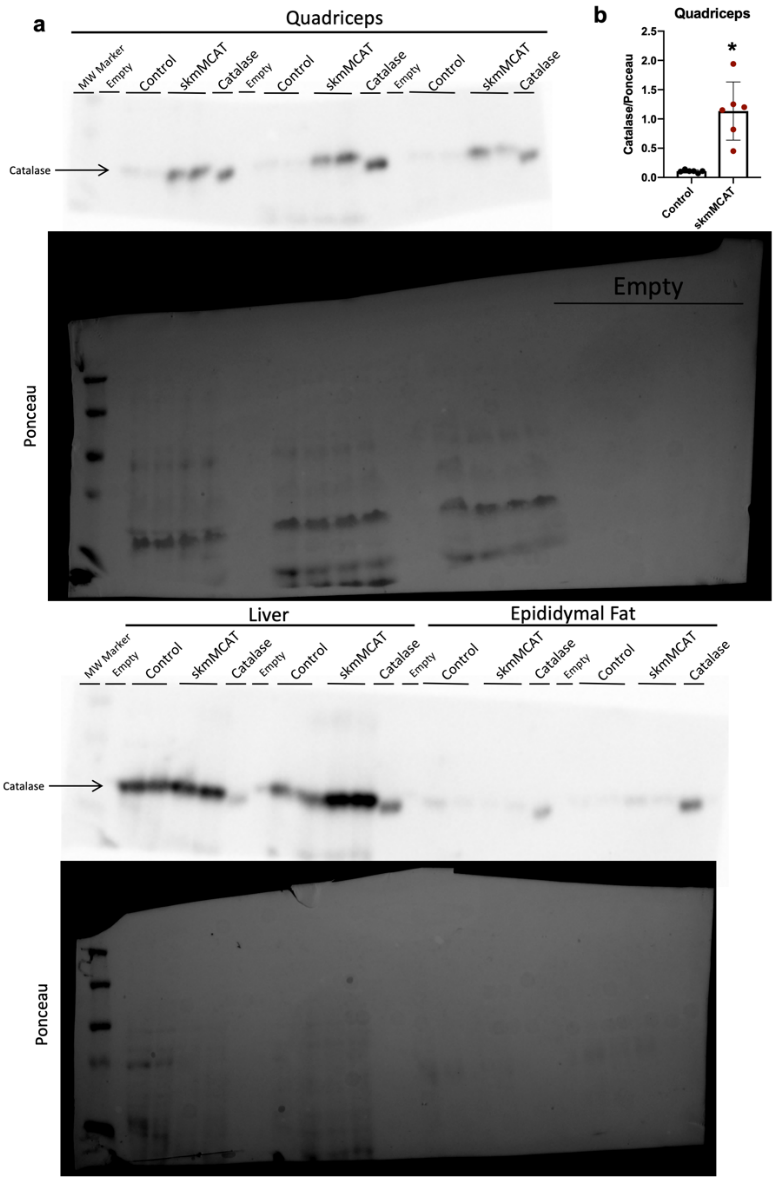

Supplemental Figure 3. skmMCAT and skmPRDX3 Overexpress the MCAT and PRDX3 transgenes. a) Representative western blots of catalase protein content in tissue homogenate of quadriceps femoris, liver, and epididymal fat and **b)** quantification of quadriceps femoris catalase protein content normalized to ponceau total protein from female wildtype and skmMCAT mice with bovine catalase loaded as a control (n = 6). **c)** Gene expression of mouse and human (MCAT) catalase by RT-PCR in control and skmMCAT gastrocnemius (n=4-5). **d)** Representative mass spectrometry peak of a peptide found in both human and mouse catalase protein in skmMCAT (top) and control (bottom) mouse gastrocnemius. **e)** Representative western blots and **f)** quantification of PRDX3 protein content in tissue homogenate of heart, gastrocnemius, and quadriceps femoris normalized to ponceau total protein from female wildtype and skmPRDX3 mice (n = 2-3). All plots represent mean ± standard deviation. Statistical significance determined by two-tailed student’s t-test (*p<0.05 for designated comparison)

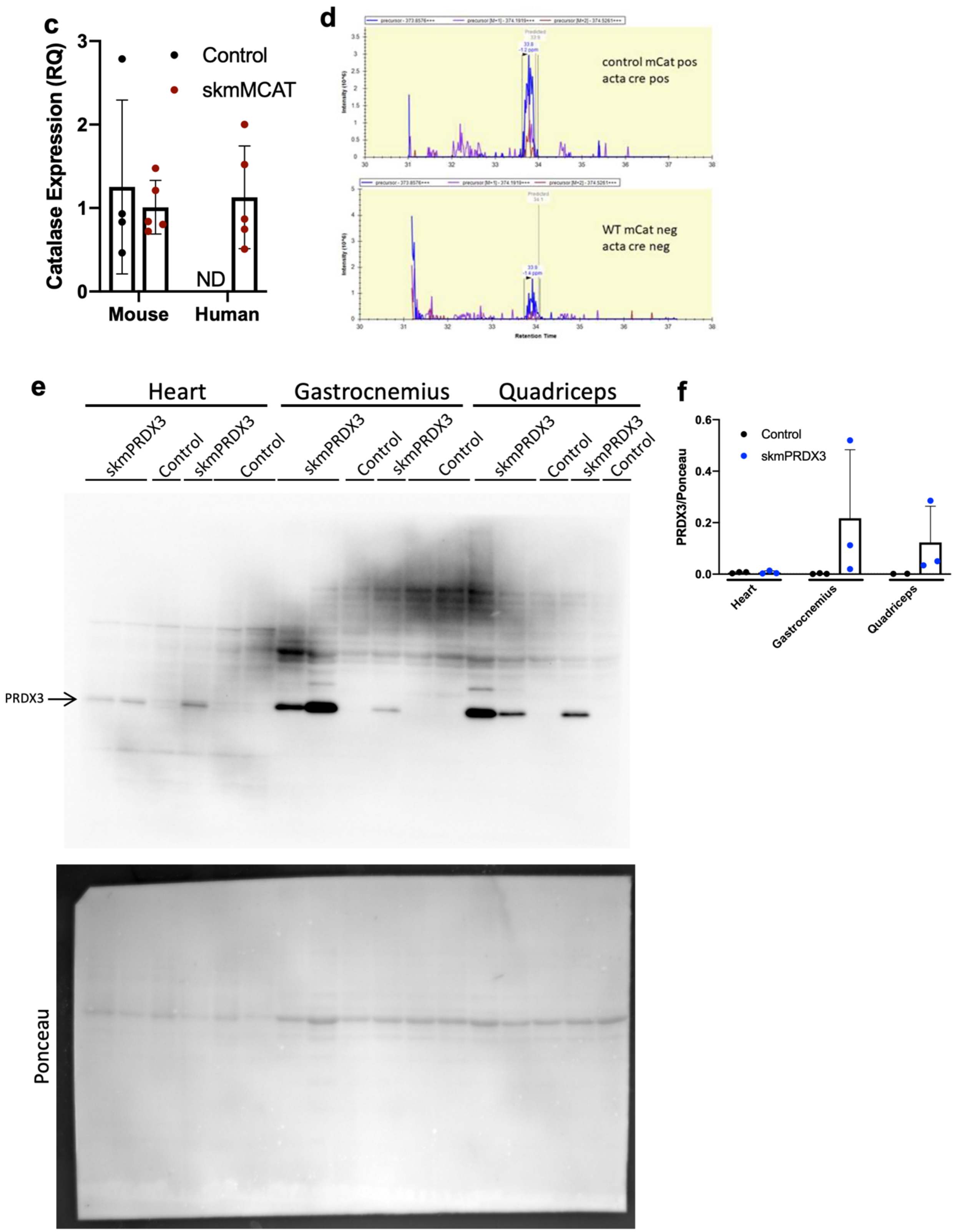

Supplemental Figure 3 Continued.

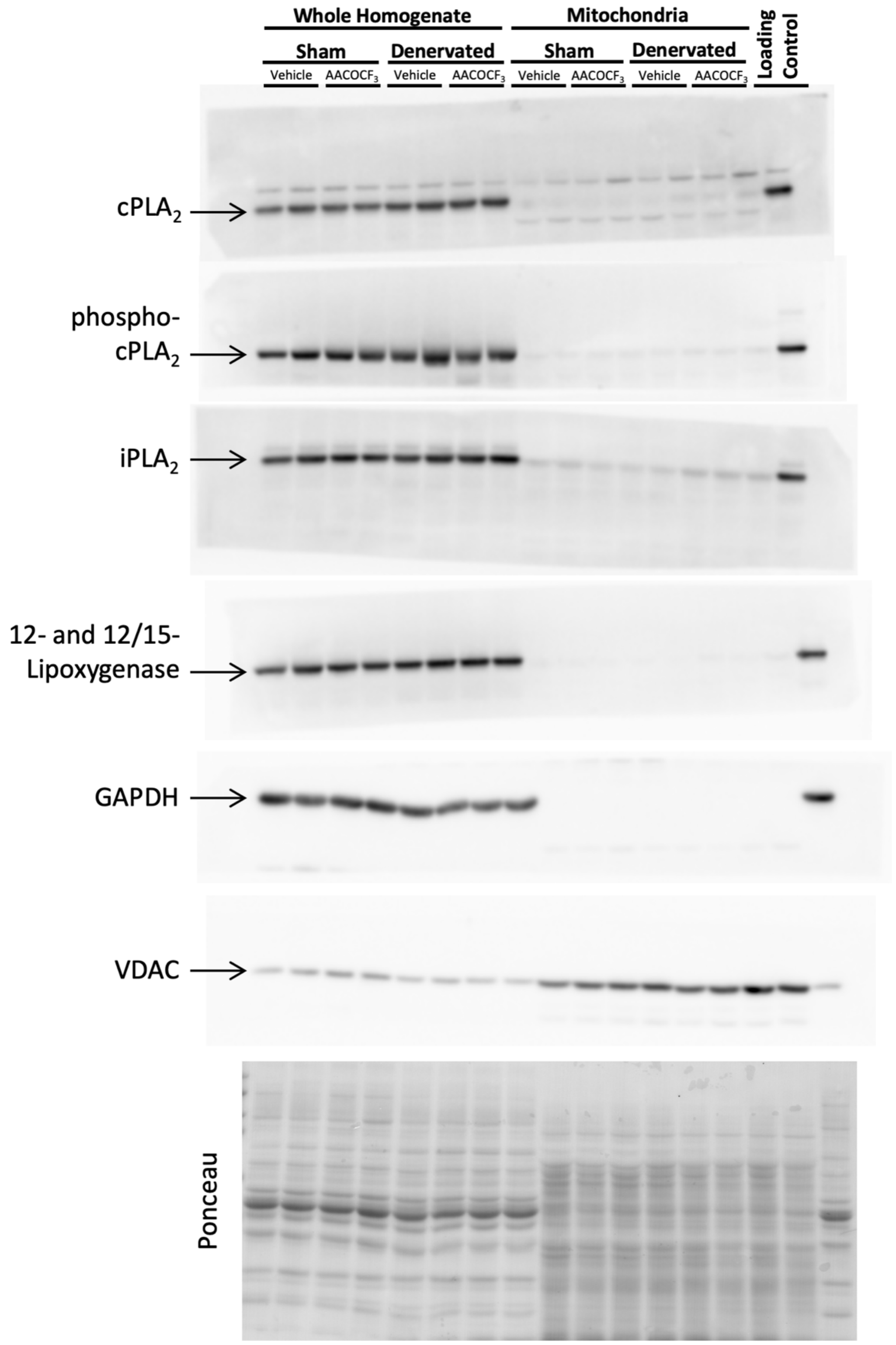

Supplemental Figure 4. Full Blot Images. Loading order and uncropped blot images for blots used in Figure 7. Blots were imaged using automatic exposure determined by G:BOX imaging platform.

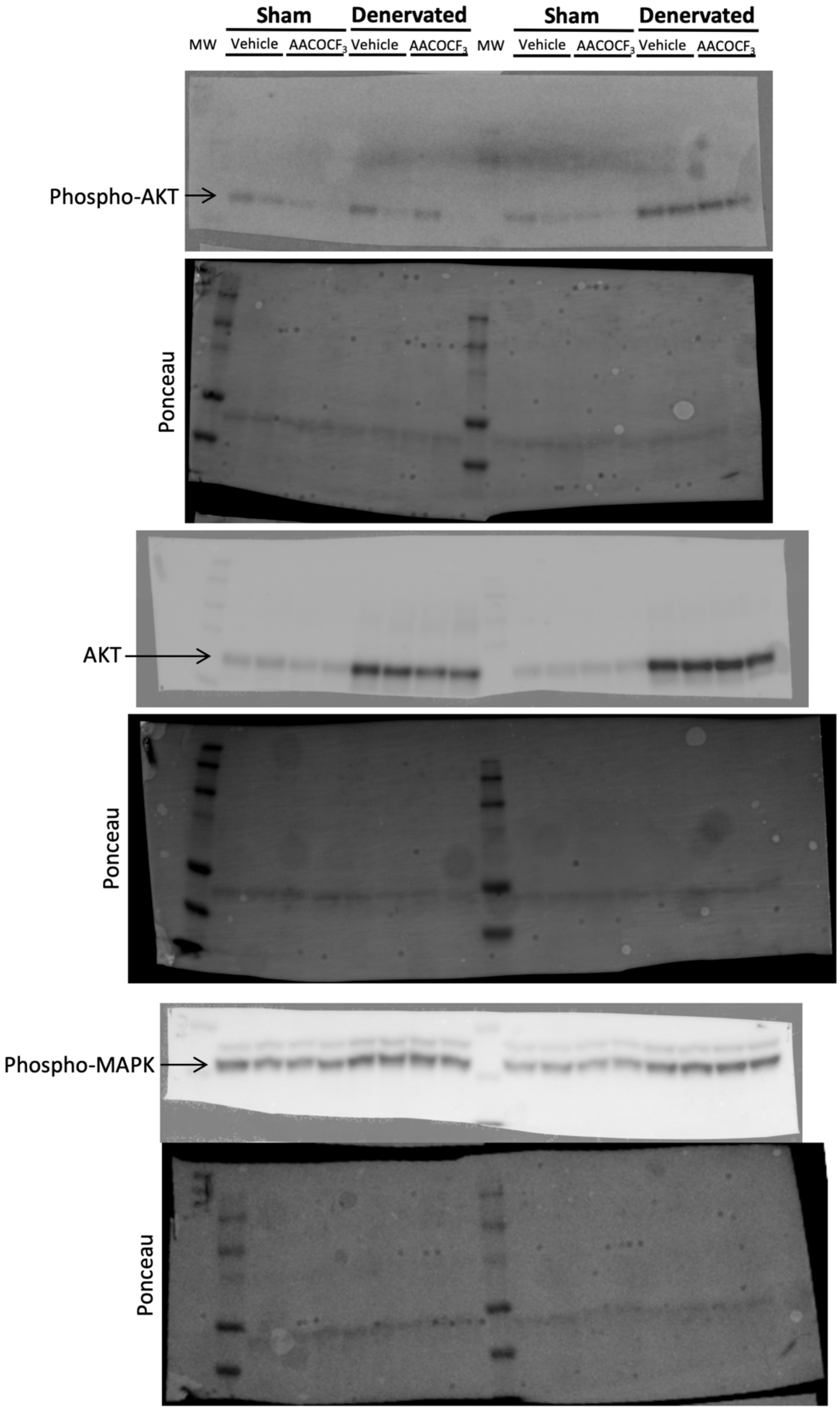

Supplemental Figure 5. Full Blot Images. Loading order and uncropped blot images for blots used in Figure 8. Blots were imaged using automatic exposure determined by G:BOX imaging platform.

Supplemental Table 1. skmMCAT Expression Did Not Significantly Affect Protein Content of Other Antioxidants or Metabolic Proteins in Gastrocnemius Muscle. Protein content (pmol protein/100 µg total protein) of metabolic and antioxidant proteins in the gastrocnemius muscle of female control and skmMCAT mice (n = 6). Catalase is a quantification of only mouse catalase. Mean ± standard deviation. Statistical significance determined by two-tailed student's t-test (*p < 0.05 compared to control) with Benjamini-Hochberg FDR correction (#q < 0.05 compared to control).

	Control			skmMCAT				
Protein	Mean		St. dev.	Mean		St. dev.	T-test	Q-Value
HSP90B1	0.12	±	0.012	0.11	±	0.010	0.738	0.996
HSPA1A	0.84	±	0.071	0.86	±	0.043	0.525	0.996
HSPA5	0.08	±	0.009	0.08	±	0.005	0.475	0.996
HSPA9	0.18	±	0.043	0.18	±	0.027	0.956	0.996
HSPD1	0.41	±	0.074	0.40	±	0.042	0.886	0.996
IDH1	0.08	±	0.014	0.07	±	0.004	0.149	0.738
IDH2	1.48	±	0.611	1.37	±	0.268	0.679	0.996
IDH3A	0.70	±	0.158	0.73	±	0.065	0.680	0.996
IDH3B	0.49	±	0.098	0.50	±	0.062	0.735	0.996
IDH3G	0.64	±	0.138	0.64	±	0.115	0.967	0.996
LDHA	20.93	±	2.085	23.52	±	1.886	0.048	0.439
LDHB	0.91	±	0.382	0.82	±	0.187	0.647	0.996
LONP1	0.05	±	0.009	0.05	±	0.007	0.894	0.996
MDH1	2.65	±	0.590	2.62	±	0.319	0.925	0.996
MDH2	3.11	±	0.618	3.22	±	0.322	0.705	0.996
MFN1	0.01	±	0.002	0.01	±	0.002	0.767	0.996
MSRA	0.04	±	0.005	0.04	±	0.005	0.944	0.996
NCL	0.03	±	0.005	0.02	±	0.002	0.353	0.98
NDUFS1	0.45	±	0.090	0.45	±	0.057	0.944	0.996
NDUFV1	0.31	±	0.058	0.31	±	0.037	0.967	0.996
OGDH	0.74	±	0.136	0.76	±	0.095	0.776	0.996
OPA1	0.03	±	0.005	0.03	±	0.003	0.987	0.996
PC	0.13	±	0.019	0.13	±	0.015	0.947	0.996
PDHA1	1.04	±	0.190	1.08	±	0.118	0.656	0.996
PDHB	0.54	±	0.087	0.61	±	0.086	0.195	0.832
PDK1	0.01	±	0.002	0.01	±	0.002	0.847	0.996
PDK2	0.10	±	0.017	0.11	±	0.016	0.697	0.996
PDK4	0.03	±	0.012	0.03	±	0.012	0.843	0.996
PECR	0.02	±	0.001	0.02	±	0.001	0.102	0.622
PFKM	4.06	±	0.357	4.41	±	0.332	0.115	0.64
PGAM2	12.22	±	1.201	14.50	±	1.576	0.018	0.304
PGK1	1.94	±	0.071	2.14	±	0.154	0.018	0.304
PHB	0.05	±	0.008	0.05	±	0.012	0.334	0.98
PHB2	0.10	±	0.019	0.12	±	0.032	0.330	0.98
PKM2	12.08	±	1.211	13.66	±	0.744	0.022	0.313
PRDX1	0.63	±	0.020	0.67	±	0.032	0.048	0.439
PRDX2	0.19	±	0.008	0.20	±	0.020	0.214	0.875
PRDX3	0.29	±	0.038	0.32	±	0.045	0.177	0.803
PRDX5	0.13	±	0.016	0.16	±	0.042	0.086	0.621
PRDX6	0.16	±	0.014	0.17	±	0.023	0.351	0.98
PRKACA	0.12	±	0.012	0.13	±	0.007	0.794	0.996
PYGB	0.00	±	0.001	0.00	±	0.001	0.030	0.349
PYGM	7.47	±	0.767	8.17	±	0.695	0.127	0.677
SAMM50	0.20	±	0.035	0.20	±	0.026	0.930	0.996
SDHA	0.50	±	0.097	0.51	±	0.062	0.760	0.996
SDHB	0.25	±	0.048	0.27	±	0.027	0.452	0.996
SDHC	0.06	±	0.006	0.06	±	0.013	0.461	0.996
SLC25A11	0.36	±	0.069	0.41	±	0.110	0.375	0.98
SLC25A20	0.15	±	0.038	0.15	±	0.029	0.989	0.996
SLC25A4	2.14	±	0.370	2.41	±	0.507	0.313	0.98
LC25A4/5/31	10.84	±	1.752	12.26	±	3.008	0.339	0.98
SLC2A4	0.08	±	0.005	0.09	±	0.014	0.392	0.996
SOD1	0.40	±	0.046	0.51	±	0.069	0.013	0.304
SOD2	0.19	±	0.038	0.21	±	0.029	0.494	0.996
SUCLA2	0.60	±	0.108	0.61	±	0.056	0.724	0.996
SUCLG1	0.25	±	0.045	0.25	±	0.029	0.844	0.996
TALDO1	0.01	±	0.001	0.01	±	0.001	0.593	0.996
TKT	0.04	±	0.010	0.03	±	0.007	0.263	0.896
TPI	4.00	±	0.253	4.54	±	0.268	0.005	0.304
TUFM	0.18	±	0.032	0.18	±	0.015	0.620	0.996
TXN1	0.10	±	0.003	0.12	±	0.019	0.036	0.384
TXNRD1	0.06	±	0.004	0.06	±	0.005	0.097	0.621
UQCRC1	1.18	±	0.220	1.36	±	0.295	0.246	0.875

	Control			skmMCAT				
Protein	Mean		St. dev.	Mean		St. dev.	T-test	Q-Value
HSP90B1	0.115	±	0.012	0.113	±	0.010	0.738	0.996
HSPA1A	0.841	±	0.071	0.863	±	0.043	0.525	0.996
HSPA5	0.084	±	0.009	0.081	±	0.005	0.475	0.996
HSPA9	0.183	±	0.043	0.184	±	0.027	0.956	0.996
HSPD1	0.409	±	0.074	0.404	±	0.042	0.886	0.996
IDH1	0.083	±	0.014	0.074	±	0.004	0.149	0.738
IDH2	1.483	±	0.611	1.367	±	0.268	0.679	0.996
IDH3A	0.697	±	0.158	0.727	±	0.065	0.680	0.996
IDH3B	0.486	±	0.098	0.503	±	0.062	0.735	0.996
IDH3G	0.641	±	0.138	0.644	±	0.115	0.967	0.996
LDHA	20.928	±	2.085	23.518	±	1.886	0.048	0.439
LDHB	0.905	±	0.382	0.823	±	0.187	0.647	0.996
LONP1	0.054	±	0.009	0.054	±	0.007	0.894	0.996
MDH1	2.647	±	0.590	2.621	±	0.319	0.925	0.996
MDH2	3.106	±	0.618	3.217	±	0.322	0.705	0.996
MFN1	0.011	±	0.002	0.011	±	0.002	0.767	0.996
MSRA	0.037	±	0.005	0.037	±	0.005	0.944	0.996
NCL	0.026	±	0.005	0.023	±	0.002	0.353	0.98
NDUFS1	0.446	±	0.090	0.449	±	0.057	0.944	0.996
NDUFV1	0.310	±	0.058	0.312	±	0.037	0.967	0.996
OGDH	0.745	±	0.136	0.765	±	0.095	0.776	0.996
OPA1	0.028	±	0.005	0.028	±	0.003	0.987	0.996
PC	0.133	±	0.019	0.134	±	0.015	0.947	0.996
PDHA1	1.040	±	0.190	1.081	±	0.118	0.656	0.996
PDHB	0.540	±	0.087	0.609	±	0.086	0.195	0.832
PDK1	0.011	±	0.002	0.010	±	0.002	0.847	0.996
PDK2	0.102	±	0.017	0.106	±	0.016	0.697	0.996
PDK4	0.030	±	0.012	0.031	±	0.012	0.843	0.996
PECR	0.017	±	0.001	0.016	±	0.001	0.102	0.622
PFKM	4.062	±	0.357	4.406	±	0.332	0.115	0.64
PGAM2	12.217	±	1.201	14.504	±	1.576	0.018	0.304
PGK1	1.944	±	0.071	2.140	±	0.154	0.018	0.304
PHB	0.047	±	0.008	0.053	±	0.012	0.334	0.98
PHB2	0.100	±	0.019	0.115	±	0.032	0.330	0.98
PKM2	12.082	±	1.211	13.659	±	0.744	0.022	0.313
PRDX1	0.633	±	0.020	0.668	±	0.032	0.048	0.439
PRDX2	0.188	±	0.008	0.200	±	0.020	0.214	0.875
PRDX3	0.285	±	0.038	0.320	±	0.045	0.177	0.803
PRDX5	0.127	±	0.016	0.163	±	0.042	0.086	0.621
PRDX6	0.162	±	0.014	0.173	±	0.023	0.351	0.98
PRKACA	0.124	±	0.012	0.126	±	0.007	0.794	0.996
PYGB	0.005	±	0.001	0.003	±	0.001	0.030	0.349
PYGM	7.465	±	0.767	8.169	±	0.695	0.127	0.677
SAMM50	0.201	±	0.035	0.203	±	0.026	0.930	0.996
SDHA	0.499	±	0.097	0.514	±	0.062	0.760	0.996
SDHB	0.251	±	0.048	0.268	±	0.027	0.452	0.996
SDHC	0.060	±	0.006	0.065	±	0.013	0.461	0.996
SLC25A11	0.363	±	0.069	0.412	±	0.110	0.375	0.98
SLC25A20	0.148	±	0.038	0.148	±	0.029	0.989	0.996
SLC25A4	2.137	±	0.370	2.410	±	0.507	0.313	0.98
SLC2A4	0.081	±	0.005	0.087	±	0.014	0.392	0.996
SOD1	0.404	±	0.046	0.507	±	0.069	0.013	0.304
SOD2	0.194	±	0.038	0.208	±	0.029	0.494	0.996
SUCLA2	0.595	±	0.108	0.613	±	0.056	0.724	0.996
SUCLG1	0.254	±	0.045	0.250	±	0.029	0.844	0.996
TALDO1	0.006	±	0.001	0.006	±	0.001	0.593	0.996
TKT	0.040	±	0.010	0.034	±	0.007	0.263	0.896
TPI	4.003	±	0.253	4.540	±	0.268	0.005	0.304
TUFM	0.176	±	0.032	0.184	±	0.015	0.620	0.996
TXN1	0.098	±	0.003	0.117	±	0.019	0.036	0.384
TXNRD1	0.060	±	0.004	0.065	±	0.005	0.097	0.621
UQCRC1	1.177	±	0.220	1.362	±	0.295	0.246	0.875

Supplemental Table 2. List of Antibodies Used in Western Blot Analysis.

Western Blots	
**Primary antibodies**	**Catalogue #**
cPLA2	Cell Signaling, 2832s
Phospho-cPLA2	Cell Signaling, 2831S
iPLA2, group VI	Santa Cruz, sc-376563
12/15-LOX	Santa Cruz, sc-133085
GAPDH	Sigma, G8795
VDAC	Cell Signaling, 4661
Phospho-Akt (Ser473)	Cell Signaling, 4060S
Akt (pan)	Cell Signaling, 4691S
p44/42 MAPK (Erk1/2)	Cell Signaling, 4695
Catalase	Athens, 01-05-030000
PRDX3	Abcam, EPR8115

Supplemental Files.

Differentially Expressed Genes.xlsx. Differentially expressed genes in RNAseq. PC (control), 24h (0.5-1 days denervation), 4d (2-4 days denervation, 7d (7 days denervation), and 14d (14 days denervation). Sample_1, Sample_2 columns specify times points compared. Genes with q_value (false discovery rate) below 0.05 were selected as differentially expressed.
Biological Processes.xlsx. Gene ontology biological process sets significantly enriched in the RNAseq differentially expressed genes, identified with GOStats package from Bioconductor. PC (control), 24h (0.5-1 days denervation), 4d (2-4 days denervation, 7d (7 days denervation), and 14d (14 days denervation).
Canonical Pathways.xlsx. Differentially regulated canonical pathways in RNAseq. PC (control), 24h (0.5-1 days denervation), 4d (2-4 days denervation, 7d (7 days denervation), and 14d (14 days denervation). Affected pathways at the different time points are displayed by Comparative Analysis in Ingenuity.

REFERENCES

1 Pharaoh, G. *et al.* Metabolic and Stress Response Changes Precede Disease Onset in the Spinal Cord of Mutant SOD1 ALS Mice. *Front Neurosci* **13**, 487, doi:10.3389/fnins.2019.00487 (2019).
